# You are fair, but I expect you to also behave unfairly: Positive asymmetry in trait-behavior relations for moderate morality information

**DOI:** 10.1371/journal.pone.0180686

**Published:** 2017-07-11

**Authors:** Patrice Rusconi, Simona Sacchi, Roberta Capellini, Marco Brambilla, Paolo Cherubini

**Affiliations:** 1 School of Psychology, University of Surrey, Guildford, Surrey, United Kingdom; 2 Department of Psychology, University of Milano-Bicocca, Milano, Italy; University of Pennsylvania, UNITED STATES

## Abstract

Trait inference in person perception is based on observers’ implicit assumptions about the relations between trait adjectives (e.g., fair) and the either consistent or inconsistent behaviors (e.g., having double standards) that an actor can manifest. This article presents new empirical data and theoretical interpretations on people’ behavioral expectations, that is, people’s perceived trait-behavior relations along the morality (versus competence) dimension. We specifically address the issue of the moderate levels of both traits and behaviors almost neglected by prior research by using a measure of the perceived general frequency of behaviors. A preliminary study identifies a set of competence- and morality-related traits and a subset of traits balanced for valence. Studies 1–2 show that moral target persons are associated with greater behavioral flexibility than immoral ones where abstract categories of behaviors are concerned. For example, participants judge it more likely that a fair person would behave unfairly than an unfair person would behave fairly. Study 3 replicates the results of the first 2 studies using concrete categories of behaviors (e.g., telling the truth/omitting some information). Study 4 shows that the positive asymmetry in morality-related trait-behavior relations holds for both North-American and European (i.e., Italian) individuals. A small-scale meta-analysis confirms the existence of a positive asymmetry in trait-behavior relations along both morality and competence dimensions for moderate levels of both traits and behaviors. We discuss these findings in relation to prior models and results on trait-behavior relations and we advance a motivational explanation based on self-protection.

## Introduction

Morality (e.g., trustworthiness, honesty, and sincerity) forms the primary basis for the general evaluation of others [1,2,3,4,5,6]. Several studies in the area of person perception have revealed that global impressions are influenced more by morality than by evidence related to other key judgment domains, such as competence or sociability [5,7] (for a review, see [1]). Furthermore, morality-related traits are paramount and are treated differently from traits pertaining to other dimensions (e.g., [8,9,10,11,12,13]). The distinctiveness of morality is also evidenced by a negativity effect that is absent in other realms (e.g., [14]). Indeed, past research has shown that people give more weight to immoral behaviors and evidence that falsifies moral traits, rather than moral behaviors and evidence that confirms morality (e.g., [2], Study 2; [15,16]).

In the present research, we further deepened this research area focusing on “the flip side of attribution” [17], namely people’s “behavior assumptions” (e.g., [18,19]). In other words, we investigated the extent of the behavioral range associated with the positive and negative poles of a trait when both traits and behaviors are moderate. Therefore, we aimed to answer the following question: When people have formed an initial impression (“an initial dispositional characterization”, [20], p. 589) of a target person (e.g., “*Homer is lazy*”), how likely are they to assume that the target person engages in trait-consistent (e.g., “*Homer is lying on the sofa”*) versus trait-inconsistent behaviors (e.g., “*Homer is working hard”*)? In other words, going beyond the path from behaviors to traits (i.e., trait inference from behaviors), we examined the path from traits to behaviors (i.e., the behavioral range associated with a trait).

More specifically, we explored behavior assumptions as a function of trait type (i.e., competence- versus morality-related traits); thus, we focused on the type and frequency of behaviors people associate with a moral or competent (versus immoral and incompetent) target person. Our hypothesis was that people would hold the implicit assumption that moral individuals are more likely than immoral persons to behave in an inconsistent way.

Since trait-behavior relations guide the controlled, correction stage of trait attribution (e.g., [20,21,22]), these asymmetries in behavioral expectations along the morality dimension might inform our understanding of the origins of judgment biases in a key domain of social perception.

### Trait-behavior relations along morality versus competence dimensions: Open questions

As noted previously, the weight assigned to the positive and negative poles of the morality-trait continuum is asymmetric. One of the explanations given for this negativity effect in the moral domain is based on people’s perceptions of trait-behavior relations, also known as “implicational schemata” (e.g., [17,18,23,24]). These schemata are implicit assumptions that people make about the range of behaviors that could be manifested at different (e.g., high, moderate, low) trait levels. Trait-behavior relations were first analyzed by Reeder and Brewer [18] and then empirically investigated in a systematic way by Reeder and colleagues [17]. In particular, Reeder and colleagues [17] specified Reeder and Brewer’s [18] analysis by considering the determinants of trait-behavior relations. Based on Heider’s [25] analysis of the constituents of an actor’s action, they asked participants about their perceptions of the attempts (what people try to do, that is, “intended variability”), ability (what people can do, “potential variability”), and frequency (what people actually, or generally, do, “general variability”) of actors’ behaviors in relation to three trait classes: morality, ability, and preference ([17], p. 359). Whereas the measure of potential variability was designed to test trait-behavior relations for competence traits, the measures of intended variability and general variability were related to trait-behavior relations for morality. Their experiments showed that both competence and morality traits were characterized by “*directional tendencies*” ([17], p. 356), or asymmetric relations between trait levels and behaviors, in keeping with the “hierarchically restrictive schema” proposed by Reeder and Brewer [18].

More precisely, a trait pertaining to competence (e.g., intelligence/unintelligent) is more behaviorally restricted at the negative extreme of its continuum than at its positive extreme. For example, Reeder and colleagues [17] found that when using the potential variability measure (designed to test the trait-behavior relations for competence traits), participants thought it unlikely that a very unintelligent person would be capable of portraying a person who is highly intelligent. In contrast, a very intelligent person was thought of being more capable of adequately portraying a very unintelligent person (see the negative slopes of dashed curves for the ability traits in Reeder et al. [17], pp. 363 and 369). Traits pertaining to the morality dimension (e.g., honesty/dishonesty) are also asymmetric in their relations with behaviors, but with a reverse pattern compared to competence-related traits. Hence, traits are more behaviorally restricted at the positive extreme of the morality dimension than at its negative extreme. For example, when using the intended variability measure (designed to test the trait-behavior relations for morality traits), Reeder et al. [17] found that participants judged it more likely that a very dishonest person would try to act very honestly, than that a very honest person would try to act very dishonestly if they were rewarded for doing so (see the positive slopes of dashed curves for the morality traits in Reeder et al. [17], pp. 363 and 369). Therefore, individuals at the negative extreme of a morality trait or at the positive extreme of a competence trait have a wider range of behaviors at their disposal than individuals at the morality positive extreme or competence negative extreme do.

**Fig 1:**
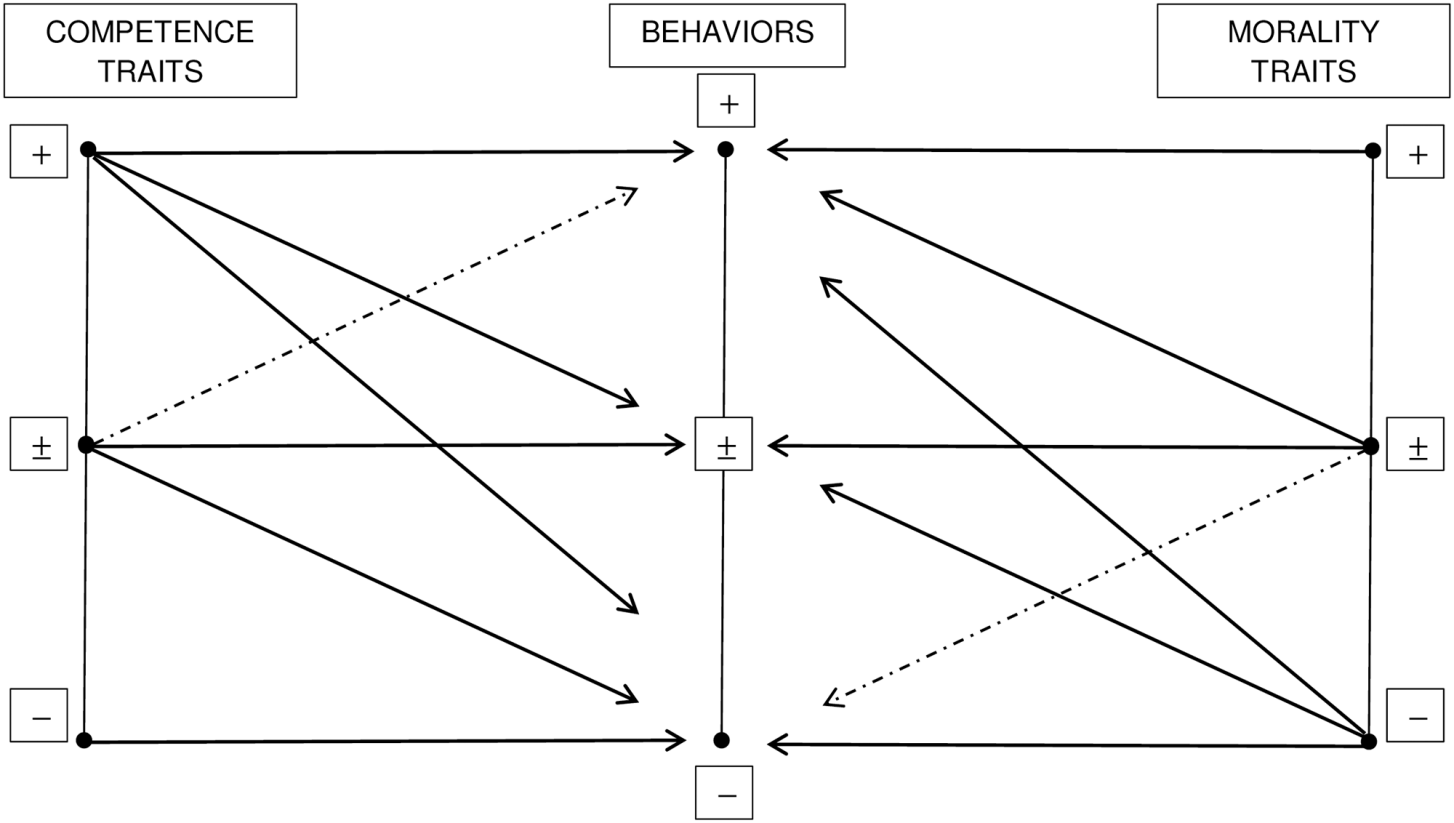
Representation of the hierarchical restrictive schema [17,18]. Positive competence- (left) and morality-related (right) traits (e.g., intelligent and honest). +, ±, and − indicate high, moderate, and low levels of traits/behaviors. Solid arrows indicate strong trait-behavior relations, whereas dashed arrows indicate weak trait-behavior relations.

The hierarchically restrictive schema describes also the implications for moderate levels of both traits and behaviors in the competence and morality dimensions. People who possess moderate traits are expected to engage in moderate trait-consistent behaviors. However, depending on the situation, moderately intelligent and moderately unintelligent people are also thought capable of very unintelligent behaviors, whereas moderately honest and moderately dishonest people are also assumed to engage in very honest behaviors ([18], p. 68). In contrast, there is a weak relation between moderate intelligence (i.e., being either moderately intelligent or moderately unintelligent) and the perceived capability of performing extremely intelligent behaviors and between moderate honesty (i.e., being either slightly honest or slightly dishonest) and highly dishonest behaviors (see Fig 1; see also [17], p. 357 and [18], p. 68).

Whether there is an asymmetry, in what direction and whether it is moderated by trait content for the *moderate* traits-*moderate* behaviors relation, is an open question that we aimed to address with the present research. This question is relevant because most of the literature on trait-behavior relations has focused on extreme levels of both traits and behaviors or on moderate-extreme (and vice-versa) relations (e.g., [17]). This focus on extreme information parallels a tendency found in the information integration literature to present participants with extremely positive or extremely negative evidence to obtain reliable manipulations ([26], footnote 2). However, since moderate traits and moderate behaviors are more frequently observed than extreme traits and behaviors in people’s everyday life, the analysis on the average levels of traits and behaviors could be crucial.

Another open question concerning trait-behavior relations relates to the inconsistent findings and theoretical interpretations obtained when the general variability measure has been used in the literature. This measure assesses the perceived general frequency of behaviors and, although it was originally thought to be relevant to trait-behavior relations for morality [17], it has also been used with reference to competence traits [16,27]. (For more details about the general variability measure and the justification of its use in the present research see S1 Text)

When either moderate traits or moderate behaviors were examined by means of the general variability measure, prior research did not reveal any clear differences between morality and competence [17]. The relation between moderate traits and extreme behaviors revealed an asymmetry whereby perceivers judged moderate persons to more frequently engage in extreme socially desirable (e.g., very moral, very skillful) than extreme socially undesirable (e.g., very immoral, very unskillful) behaviors; this effect was of similar magnitude for morality than for competence ([17], Experiment 1). However, the relation between extreme traits and moderate behaviors did not reveal any asymmetries or clear-cut differences between morality and competence ([17], Experiment 2).

Skowronski and Carlston used a measure similar to the general variability measure and tested moderate morality- and competence-related traits (“honest” and “dishonest” for morality, “intelligent” and “stupid” for competence) in relation to both moderate and extreme morality-related and competence-related behaviors [16]. The findings concerning trait-inconsistent behaviors were collapsed over the *moderate* and *extreme* levels of behaviors (see [16], p.693). However, the domain by valence interaction they found matched the pattern of the hierarchically restrictive schema for *extreme* levels of both traits and behaviors and for the *moderate* trait-*extreme* behavior relations. The only comparison between moderate and extreme levels of behaviors revealed that perceivers judged it more likely that a person with a moderate trait would emit moderate rather than extreme trait-inconsistent behaviors.

More recently, Tausch, Kenworthy, and Hewstone [27] investigated the perceived likelihood of engaging in trait-inconsistent behaviors (a measure similar to the general variability measure, which they called “diagnosticity” [27], p. 550) along competence- and warmth-related moderate traits (e.g., “trustworthy”, “intelligent”). They used a procedure which mimicked Rothbart and Park’s [28] one, a method that most likely led participants to think about moderate behaviors ([27], p. 548). Again, as suggested by the results in Skowronski and Carlston’s [16] study, Tausch et al.’s [27] results were consistent with the description of the hierarchically restrictive schema for *extreme* levels of both traits and behaviors and for the *moderate* trait-*extreme* behavior relations rather than for moderate levels of both traits and behaviors.

Thus, an account of moderate levels of trait-behavior relations underlying morality (versus competence) traits that uses a measure of general variability [17] and that could reconcile these theoretical and empirical inconsistencies in the literature is still needed.

### The positive asymmetry hypothesis for moderate morality traits

Some theories and findings in the literature are supportive of a positive asymmetry in trait-behavior relations for moderate, morality-related information. Studies in the 1970s have shown that trait adjectives can be represented as distributions and negative traits have narrower dispersions than neutral and positive traits (e.g., [29,30,31,32]). These findings suggest that immoral people could have a narrower range of behaviors than moral people could. Rothbart and Park [28] found that traits such as “intelligent” and “honest” require many instances to confirm and few instances to disconfirm ([28], p. 138). This finding suggests that traits related to competence and morality might share some structural asymmetries. In particular, concerning the trait-behavior relation asymmetry, perceivers might require many instances before they describe a target person with a positive trait because they might assume that targets with a positive trait engage more likely in trait-inconsistent behaviors than do targets with a negative trait.

Empirical evidence from information integration studies on morality judgments also supports a positive asymmetry in trait-behavior relations at moderate levels of morality-related information. Wojciszke and colleagues [26] found a negativity effect in trait inferences and likeability evaluations of a target person when participants were given two extremely positive behaviors (e.g., “Having noticed that a shop assistant gave him too much change, Paul returned the surplus”) and two extremely negative behaviors (e.g., “Paul caused an accident and ran away”) describing the target and pertaining to morality-related traits (e.g., “honest” vs. “dishonest”). However, this effect disappeared in the condition in which participants received moderately positive (e.g., “Paul told the examiner that he did not know the answer to a question on the exam”) and negative (e.g., “Paul pushed himself to the head of a long queue”) behaviors. As discussed by Wojciszke and colleagues, these findings concerning morality-related information are in keeping with results of other information integration studies on morality judgments by Birnbaum ([33], Fig 1A) and Skowronski and Carlston ([16], Experiment 2).

### The present research

Based on the reviewed literature, we hypothesized that people would think it more likely that a moral social target would engage in trait-inconsistent behaviors (i.e., a moral person acting immorally) than that an immoral target would emit trait-inconsistent behaviors (i.e., an immoral person acting morally). In other words, we expected a positive asymmetry for the moderate traits and behaviors along the morality dimension when using a measure similar to the general variability measure [17]. This hypothesized positive asymmetry with regard to morality traits is in line with prior research that showed that people tend to question others’ morality (e.g., [2,34]). Thus, people would prefer to risk committing commission errors by considering a moral person as being able to behave also immorally rather than committing omission errors in which a moral behavior would be expected from an immoral person. A parallel motivational argument cannot be made based on the reverse assumption. Indeed, assuming that an immoral person is able to behave in a moral fashion is not as self-protecting as the reverse assumption that a moral person is able to behave immorally. This view dovetails with the finding by Trafimow and Schneider ([35], Experiment 2) that a single dishonest behavior induces most people to describe the actor as dishonest (see also [31,33]). Moreover, Meindl and colleagues [34] have recently shown an “immoral assumption effect”, whereby people are more prone to make negative morality-related trait attributions (“trait assumptions”) based on a target’s immoral behavior rather than unsociable trait attributions after a cold behavior. This difference did not hold for positive assumptions. Our hypothesis of narrower behavioral ranges associated with negative vs. positive traits for morality is consistent with range theories of information integration in impression formation by Wyer [29] and Birnbaum [30]. Indeed, “it is less likely for a person possessing a dislikable trait to be likable than for a person with a likable trait to be dislikable.” ([31], p. 560)

Furthermore, our hypothesis builds on Rothbart and Park’s [28] perspective according to which confirmation of positive traits is hard and disconfirmation is easy for both morality and competence. According to the authors, frequency is not the only factor likely to influence the ease of trait dis/confirmability [28]. In particular, a crucial variable is trait favorability. Whereas positive traits are easy to lose and difficult to acquire, negative traits are easy to acquire but difficult to lose. Furthermore, once a trait has been established and must be confirmed or disconfirmed (as at the correction stage), it is generally easier to imagine negative behaviors that disconfirm positive traits than positive behaviors that disconfirm negative traits. Moreover, there are generally more occasions for disconfirming positive traits than negative traits ([28], p. 137). These findings led us to hypothesize that people would think it more likely that trait-inconsistent behaviors are emitted by an actor possessing a positive rather than a negative trait related to the morality dimension. We expected a positive asymmetry in trait-behavior relations also for moderate competence-related traits. The hypothesized positivity along the competence dimension is based on the aforementioned finding that both “intelligent” and “honest” require many instances to confirm and few instances to disconfirm ([28], p. 138) and the positivity in competence impressions based on moderate behaviors found in information integration [26].

### Studies overview

To investigate the hypothesis that moral people would be more likely expected to engage in trait-inconsistent behaviors than immoral people for moderate levels of both traits and behaviors, we conducted four studies based on a pretest described in S2 Text and S1 Table. The studies have been approved by the University of Milano-Bicocca Ethics Committee (Prot. N. 0025855/13), in accordance with the Declaration of Helsinki and the Oviedo Convention. For all the studies, participants were properly instructed about the nature of the tasks and the general aims. Participants provided their informed consent to participate in the electronic form when not possible in other ways (i.e., when the task was administered online), and this procedure was approved by the ethics committee. In Study 1, we addressed the issue of perceived behavioral range associated with moderately positive versus negative traits using abstract categories of behavior and requiring probability estimates (in the form of percentages) from participants. We used a restrictiveness index whereby the probability of trait-inconsistent behavior for the negative trait pole was subtracted from the same probability for persons with a positive trait. In Study 2, we examined the same process by changing two methodological properties. First, we used a frequency format instead of a probability format because people judge frequencies more easily than probabilities (e.g., [36]). Second, we used a restrictiveness index that incorporated participants’ estimates of the unconditional likelihood of behaviors. Study 3 considered concrete categories of behavior. In a pretest described in S5 Text, we asked participants to produce a series of trait-inconsistent behaviors for each pole of a trait continuum, and we analyzed the number of produced behaviors. In Study 3, we used the pretested set of behaviors to ask participants about the frequency with which people would engage in these behaviors. In Study 4, we tested the hypothesis that the effects found in the previous four studies would hold cross-culturally. Accordingly, we asked both Italian and US participants to estimate the likelihood of trait-inconsistent behaviors. Finally, we conducted a small-scale meta-analysis to test the findings’ reliability.

## Study 1

In Study 1, we investigated participants’ perceptions of moderate levels of trait-behavior relations for traits involving competence and morality. For each of the pretested traits (see S2 and S3 Text), we asked participants to estimate the probability that an actor possessing a trait (e.g., honesty) would manifest trait-inconsistent behaviors (e.g., dishonest behaviors).

## Method

### Participants

Forty-eight Italian undergraduate students (35 female, 13 male, *M*_age_ = 21.96, *SD*_age_ = 2.04, range: 19–29 years) volunteered to participate in the study.

### Materials and procedure

Participants were given a booklet. On the first page, they were told that the investigation concerned “information search in social contexts”. They were also asked to provide their demographic details. On the second page, they were instructed as follows: “In the following pages, you will be presented with a series of questions concerning some characteristics. Your task is to read and answer all of the questions by marking with an ‘X’ the box with a percentage between 0 (which indicates ‘totally unlikely’) and 100 (‘totally likely’) that best corresponds to your judgment. Please answer all of the questions in this questionnaire”. A series of 46 questions followed, two for each pretested trait, each followed by an 11-point scale ranging from 0% (*totally unlikely*) to 100% (*totally likely*). Participants were required to provide probability estimates that a person with a trait would exhibit trait-inconsistent behaviors. First, for each pretested trait, participants evaluated the probability that a target person with a positive trait (e.g., an honest/intelligent person) would exhibit opposite behaviors (e.g., dishonest/stupid behaviors). Then, the participants estimated the probability of the reverse pattern. That is, they estimated the probability that a target person with a negative trait (e.g., a dishonest/stupid person) would manifest positive behaviors (e.g., honest/intelligent behaviors). To control for order effects, we devised a second version of the questionnaire with the trait presentation in the reverse order. Two questions presented to participants for the morality dimension were: “How likely do you consider it that an honest person would behave in a dishonest fashion?” and “How likely do you consider it that a dishonest person would behave in an honest fashion?” Two sample questions for the competence domain were: “How likely do you consider it that an intelligent person would behave in a stupid fashion?” and “How likely do you consider it that a stupid person would behave in an intelligent fashion?” This question phrasing is similar to the one used by Tausch et al. (Study 3) [27] for their “diagnosticity” measure (see also S1 Text). Our method does not consider instances of extreme traits and behaviors (i.e., the likelihood of *highly* honest people to engage in *very* dishonest behaviors) in a similar way to Rothbart and Park’s [28] and Tausch et al.’s [27] methods ([27], p. 548).

## Results

We computed the difference between the two probability estimates provided by participants for each trait to obtain a restrictiveness index (*R*):

R=p(¬D|H)−p(D|¬H), (1)

where *p* (means “probability of”, | represents “given that”, ¬ is the logical symbol for negation, *D* represents the positively valenced behavioral evidence, ¬*D* is the negatively valenced behavioral evidence, and *H* represents the hypothesized trait. Consider, for example, the honest/dishonest trait. We computed *R* as the difference between the participant’s probability estimate of observing dishonest behaviors, ¬*D*, from an honest person, *H*, and the participant’s probability estimate of observing honest behaviors, *D*, performed by a dishonest person, ¬*H*.

*R* is positive when the person possessing the positively valenced trait (e.g., honesty) is thought to have at her disposal a wider range of behaviors (i.e., she might also behave counter to her disposition) than the person with the opposite trait (e.g., dishonesty). Vice versa, *R* is negative when the person with a negative trait (e.g., a dishonest person) is expected to manifest a wider range of behaviors than the person with a positive trait does (e.g., an honest person). Finally, *R* is zero when persons with opposite traits have equal probabilities of exhibiting behaviors contrary to their traits.

**Fig 2:**
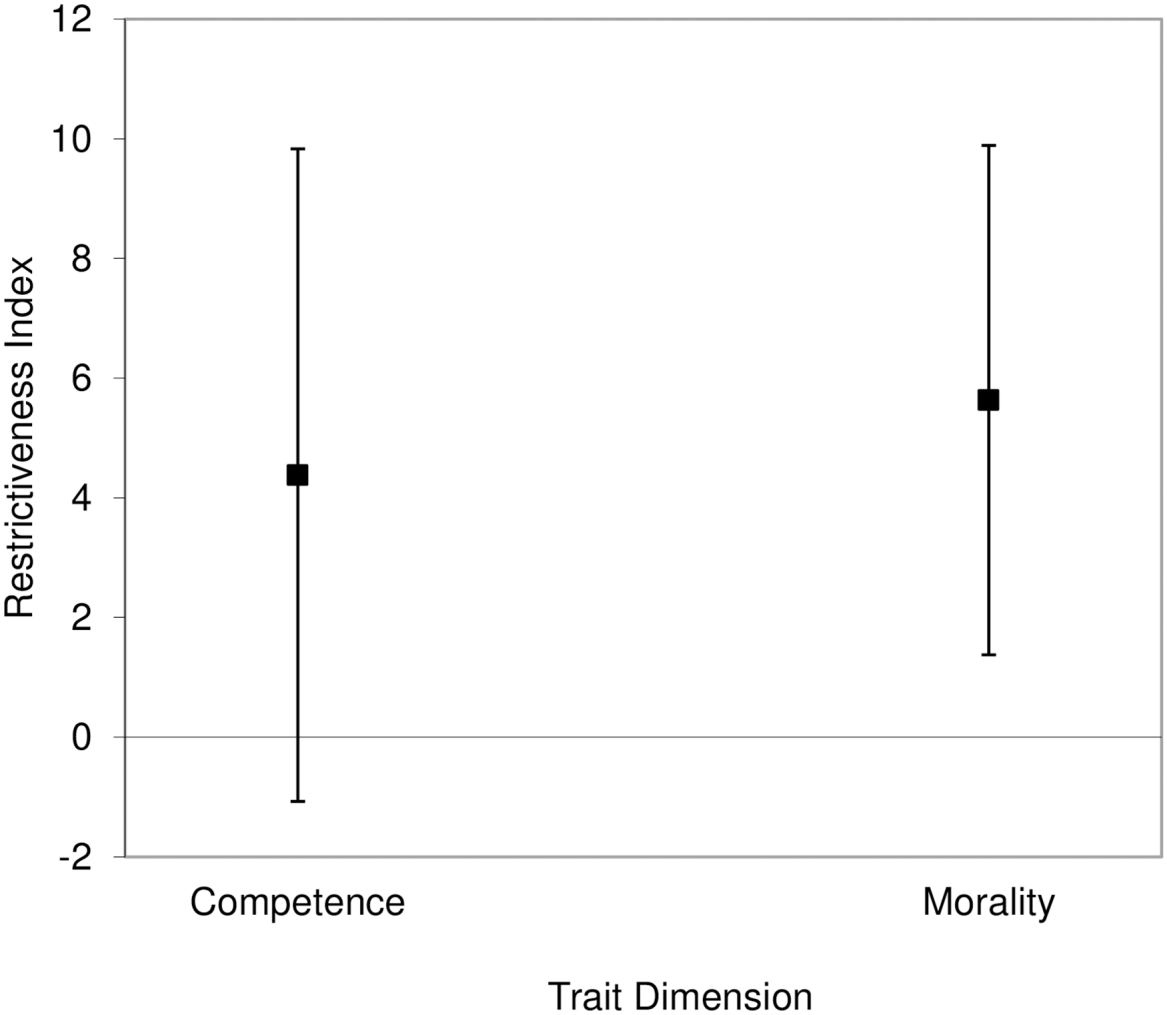
Results of the analysis on the larger set of traits in Study 1. The difference between the likelihoods of trait-inconsistent behaviors for the positive and negative poles of traits (restrictiveness index) was significantly positive for morality-related but not competence-related traits. Error bars represent 95% confidence intervals.

We averaged the eight competence’s *R*s because they were highly consistent (Cronbach’s α = .89). For the same reason, we combined the *R*s of the 15 morality-related traits (Cronbach’s α = .88). We then compared both the competence and the morality *R* indexes with zero, which is the value indicating symmetry between the two opposite poles of a trait, using one-sample *t*-tests. Furthermore, we compared the competence and morality *R* indexes to each other using a paired *t*-test. For each of the three *t*-tests, we used adjusted alpha levels of .0167 according to the correction for multiple testing illustrated by Benjamini and Hochberg [37]. We found that the competence index (*M* = 4.38, *SD* = 19.28), 95% CI of the difference [-1.22, 9.97], was not significantly different from zero, *t*(47) = 1.57, *p* = .123, *d* = .23. Furthermore, the difference between the morality index (*M* = 5.63, *SD* = 15.05), 95% CI of the difference [1.26, 9.99], and zero was significant, *t*(47) = 2.59, *p* = .013, *d* = .37. These results indicate that participants perceived the two poles of competence-related traits as having equal behavioral flexibility. In contrast, people with positive morality-related traits were perceived as having a wider range of behaviors than people with negative traits (Fig 2).

Finally, we did not find a significant difference between the two *R* indexes (95% CI of the difference [-5.69, 3.19]), *t*(47) = -.57, *p* = .574, *d* = .08. (Cohen’s *d* was corrected for the dependence between means as illustrated by Morris & DeShon, Equation 8 [38]. We shall refer to this correction as Cohen’s corrected *d* in the subsequent analyses).

### Controlling for trait valence

Some confounds might have muddied the previous comparisons between competence-related and morality-related traits. They both relate to trait valence, which is a crucial dimension in trait attribution (e.g., [28,39,40]). First, competence traits could have been more polarized than morality traits or vice versa at either the positive or the negative pole of the traits. Second, within each domain, traits could have been biased to yield an asymmetry between the positive and the negative poles of the traits. For example, immoral traits could have been valued as more polarized than moral traits, potentially accounting for the finding that immoral target persons were judged to be more behaviorally restricted (i.e., unlikely to perform moral behaviors) than moral target persons (who were also likely to exhibit immoral behaviors). In S3 Text, we report the analyses we conducted to control for these possible confounders. We found that the following traits were balanced for valence: intelligent/stupid, efficient/inefficient, and competent/incompetent for the competence domain and righteous/unrighteous, sincere/insincere, and fair/unfair for the morality domain. Although we did not use the adverb “very” that could have conveyed the extremity of traits, we confirmed that the selected traits were perceived as moderate by analyzing the participants’ valence ratings (see S3 Text).

### Analyses on the trait subsets balanced for valence

Once we identified the competence and morality traits that were sufficiently balanced for valence, we reanalyzed the data. For each of the five *t*-tests we will present in this subsection, we used adjusted significance levels of .0200 following Benjamini and Hochberg’s method [37]. *R* was not highly consistent across the three competence traits (i.e., intelligent/stupid, efficient/inefficient, and competent/incompetent), Cronbach’s α = .58. Therefore, we considered each competence trait separately. We performed three one-sample *t*-tests to compare *R* with zero, that is, the value of trait symmetry. We found that all of the competence traits had a positive *R*, but only the value of *R* relative to the intelligent/stupid disposition was significantly higher than zero (Table 1).

**Table 1:** Study 1: Results of one-sample t-tests. Comparisons of the restrictiveness indexes (R) with zero (that indicates symmetry of restrictiveness) for the competence-related traits balanced for valence.

Trait	Mean (*SD*)	95% CI of the Difference	*t*-value (df)	*p*-value	Cohen’s *d*
Intelligent/stupid	18.33 (24.35)	[11.26, 25.4]	5.22 (47)	< .001	.75
Efficient/inefficient	3.96 (23.22)	[-2.78, 10.7]	1.18(47)	.244	.17
Competent/incompetent	2.71 (23.59)	[-4.14, 9.56]	.8 (47)	.430	.11

*Note*. CI = confidence interval.

*R* was consistent across the three morality traits (i.e., righteous/unrighteous, sincere/insincere, and fair/unfair), Cronbach’s α = .72. Therefore, we averaged the three traits’ values and compared the obtained *R* value with zero using a one-sample *t*-test. We found that *R* (*M* = 7.43, *SD* = 18.45), 95% CI of the difference [2.07, 12.79], was significantly higher than zero, *t*(47) = 2.79, *p* = .008, *d* = .4.

Finally, we compared the *R* values of competence- and morality-related traits (each collapsed across the three traits) by means of a paired *t*-test. We found that *R* did not differ significantly whether the traits related to competence (*M* = 8.33, *SD* = 17.52) or morality (*M* = 7.43, *SD* = 18.45), 95% CI of the difference [-6, 4.19], *t*(47) = -.36, *p* = .723, Cohen’s corrected *d* = .05.

## Discussion

Study 1 revealed that the traits along the morality/immorality dimension were perceived as positively asymmetric. The positive poles of the moral traits were perceived as more behaviorally unrestricted than the negative ones. In other words, the participants assumed that individuals with a positive morality-related trait would engage in trait-inconsistent behaviors to a greater extent than individuals with a negative trait would do. This finding held both when considering a large set of traits and when analyzing a subset of these traits pretested for valence.

Overall, we found that competence-related traits were perceived as having equal behavioral flexibility at the opposite poles of the traits. Indeed, the *R* index was not significantly different from zero, which indicated trait symmetry. The exception concerned the intelligent/stupid trait, which was perceived as positively asymmetric. The comparison between the *R* indexes of competence and morality revealed that the two dimensions did not differ significantly in terms of restrictiveness.

## Study 2

An important aspect of Study 1 is that the participants might have found it difficult to express their judgments in terms of probabilities, and this difficulty might have partly accounted for the results. Indeed, past research has shown that people might find it easier to receive input and produce output in the form of frequencies rather than probabilities (e.g., [36,41]). Therefore, in Study 2, we changed the question phrasing used in Study 1 by replacing the probability (in the form of percentages) with a frequentist question. This question phrasing is closer to that used by Reeder et al. [17] to assess “general variability” ([17], p. 361) compared with the one used in Study 1. Furthermore, we took into account the base rate of behaviors, as we shall explain in the following section.

## Method

### Participants

Fifty-two Italian undergraduate students (36 female, 16 male, *M*_age_ = 22.87, *SD*_age_ = 4.41, range: 19–51 years) volunteered to participate in the study.

### Materials and procedure

Materials and procedure were the same as in Study 1 except as follows. The participants were asked to estimate how many times, out of 10 behaviors, a person with a trait would manifest trait-inconsistent behaviors. For example, participants were asked, “Out of 10 behaviors, how many times do you think that an honest person would behave in a dishonest fashion?” Then, they were asked the reverse: “Out of 10 behaviors, how many times do you think that a dishonest person would behave in an honest fashion?” Participants were asked to mark with an “X” a number between 0 and 10. We devised four versions of the questionnaire to control for order effects. Therefore, we varied the trait presentation order (the original one versus the reverse one) and the question presentation order (the first judgment was about a person with a positively valenced disposition versus a negatively valenced disposition).

In addition, at the end of the questionnaire, the participants were required to provide an estimate of the prevalence of each trait. For example, they were asked: “Out of 10 persons, how many do you consider honest?” Again, the participants were required to mark with an “X” a number between 0 and 10. We did not ask about the prevalence of the negatively valenced dispositions because we assumed that their prevalence was complementary to the prevalence of the positive trait ends. Therefore, there were 23 trait-prevalence questions. As we shall explain in the Results section, this additional request from the participants, compared with the task requirement in Study 1, allowed us to compute the frequency of behavior, which is a factor that influences trait attribution (e.g., [28]). Reeder and colleagues ([19], p. 42; [20], p. 587) argued that a base-rate principle underlies trait-behavior relations along morality traits rather than along competence traits. We argue that base-rate considerations are equally relevant for competence and morality traits and that it is reasonable to incorporate the base-rate principle in the computation of a restrictiveness index for both competence and morality traits.

## Results

We used the estimates of trait prevalence among people (i.e., the responses to the 23 final questions of the questionnaires) to obtain a modified restrictiveness index. In doing so, we converted all of the participants’ frequency estimates into probabilities by dividing them by 10. Thus, the scale of the obtained values ranged from 0 to 1, and we could straightforwardly apply a Bayesian analysis (for the use of this type of analysis in a social hypothesis-testing study, see, e.g., [42]; see also [43,44]). Trait-prevalence estimates correspond to prior probabilities under a Bayesian perspective, that is, to *p*(*H*) where *H*, in our questions, was the number of persons deemed to possess a particular trait. From prior probabilities, one can derive the probability of occurrence of behaviors, that is, *p*(*D*), by means of the following equation (e.g., [42], p. 248):

p(D)=p(D|H)×p(H)+p(D|¬H)×p(¬H), (2)

where *p*(*D*|*H*) stands for the probability of observing a new datum, *D*, given the truth of the hypothesis under consideration, *H*. For example, this could be the probability of an honest behavior, *D*, being performed by an honest person, *H*. Therefore, this term was the complement of the participants’ estimate of how often a dishonest behavior, ¬*D*, was performed by an honest person, *H*. We derived *p*(*D*|*H*) from the participants’ estimates using the following equation:

p(D|H)=1−p(¬D|H). (3)

The other terms in (Eq 2) are trait prevalence (that is, the prior probability that a person has a particular disposition, *p*(*H*)), and its complement, *p*(¬*H*) = 1 − *p*(*H*) (that is, the probability that a person does not have a particular disposition). Finally, *p*(*D*|¬*H*) represents the probability of observing a new datum given the falsity of the hypothesis under consideration, such as the probability of an honest behavior, *D*, displayed by a dishonest person, ¬*H*.

*p*(¬*D*) was computed as the complement of *p*(*D*) using the following equation (see, e.g., [42], p. 248):

p(¬D)=1−p(D). (4)

We analyzed the participants’ estimates of trait prevalence among people, that is, *p*(*H*), and the derived probabilities of occurrence of behaviors, that is, *p*(*D*). The findings were consistent with the negative assumptions about other people’s morality found by Meindl and colleagues [34]. Participants’ estimates of the number of people possessing a positive morality-related trait, were significantly lower than *p* = .05, whereas the difference from the chance level was not significant for positive competence-related traits. The probabilities of occurrence of positively valenced behaviors that we derived in the way explained above were also significantly lower than the *p* = .05 for the morality dimension, but not for the competence dimension. These results held both when considering the large set of morality- and competence-related traits and when analyzing the subsets of traits that were balanced for valence (see S4 Text for a detailed description of these findings).

Furthermore, we could compute a revised *R* index that weighted the terms used in (Eq 1) by the values of Eqs (2) and (4), that is, *p*(*D*) and *p*(¬*D*). The resulting index was

$$R_{r}=\left|p\left(\neg D\right)-p\left(\neg D|H\right)\right|-\left|p\left(D\right)-p\left(D|\neg H\right)\right|,$$ (5)

the difference between a participant’s estimate that a person with a positively valenced trait would manifest trait-inconsistent behaviors, *p*(¬*D*|*H*), and the estimate that a person with a negatively valenced trait would behave counter to it, *p*(¬*D*|*H*), each weighted by the probability of the occurrence of the trait-inconsistent behaviors (a similar index in the context of asymmetric information search was used by Sacchi, Rusconi, Russo, Bettiga, & Cherubini [45]). Again, as for the *R* index in (Eq 1), *R*_*r*_ yields positive or negative values as a function of whether the frequency of trait-inconsistent behaviors is judged higher for the positive or the negative trait pole, whereas it is equal to zero if there is no difference between the positive and the negative trait pole.

**Fig 3:**
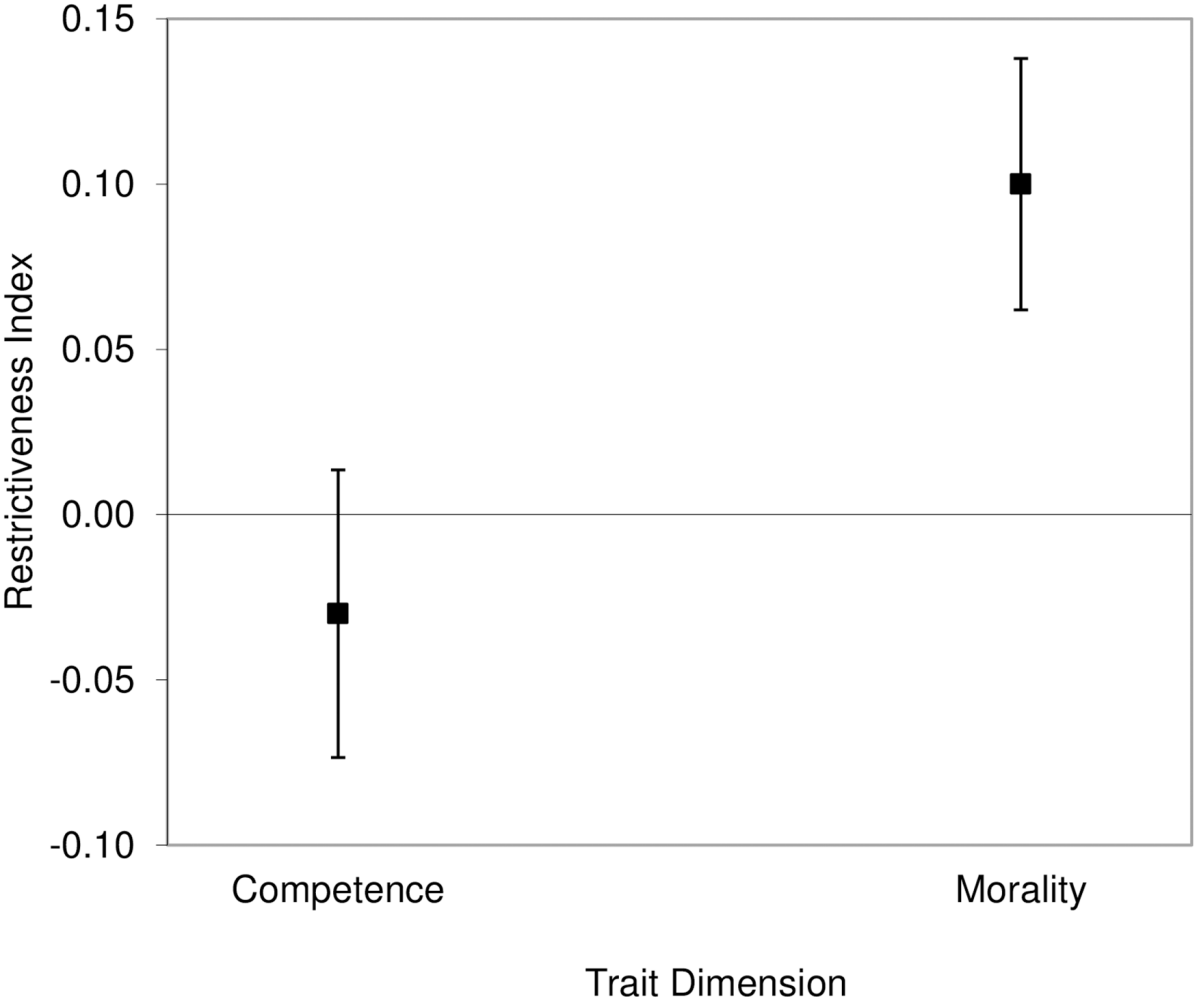
Results of the analysis on the larger set of traits in Study 2. The difference between the likelihoods of trait-inconsistent behaviors for the positive and negative poles of traits (restrictiveness index) was significantly positive for morality-related but not competence-related traits. Error bars represent 95% confidence intervals.

We averaged the *R*_*r*_ indexes of the eight competence-related traits (Cronbach’s α = .88), and the 15 morality-related traits (Cronbach’s α = .91). Using one-sample *t*-tests, we then compared both the competence and the morality *R*_*r*_ indexes with zero to determine their degree of restrictiveness. We also compared the competence *R*_*r*_ index with the morality *R*_*r*_ index using a paired *t*-test. For each of these three *t*-tests, we used corrected significance levels of .0333 according to Benjamini and Hochberg’s method [37]. We found that the competence *R*_*r*_ index (*M* = -.03, *SD* = .16), 95% CI of the difference [-.07, .02], was not significantly different from zero, *t*(51) = -1.16, *p* = .250, *d* = .16. In contrast, the morality *R*_*r*_ index (*M* = .1, *SD* = .14), 95% CI of the difference [.06, .14], was significantly higher than zero, *t*(51) = 5.03, *p* < .001, *d* = .7. Finally, there was a significant difference between competence and morality (95% CI of the difference [-.17, -.08]), *t*(51) = -6.01, *p* < .001, Cohen’s corrected *d* = .87, indicating that the morality-related traits were overall more positively asymmetric (with the positively valenced pole of the trait continuum being more behaviorally unrestricted than the negatively valenced one) than the competence-related traits (Fig 3).

### Analyses of the trait subsets balanced for valence

Following the same procedure used in Study 1, we performed the same analyses using *R*_*r*_ on the subsets of three competence-related and three morality-related traits that were balanced for valence. For each of the following three *t*-tests, we used adjusted alpha levels of .0333 after Benjamini and Hochberg’s correction [37]. Cronbach’s alphas for the *R*_*r*_ of the three competence traits, .77, and the *R*_*r*_ of the three morality traits, .73, were sufficiently high to allow us to combine the three traits of each domain. The one-sample *t*-tests comparing each collapsed variable with zero showed that the competence *R*_*r*_ index (*M* = -.02, *SD* = .17), 95% CI of the difference [-.06, .03], was not significantly different from zero, *t*(51) = -.76, *p* = .452, *d* = .1. In contrast, the morality *R*_*r*_ index (*M* = .07, *SD* = .15), 95% CI of the difference [.03, .12], was significantly higher than zero, *t*(51) = 3.51, *p* = .001, *d* = .49.

**Fig 4:**
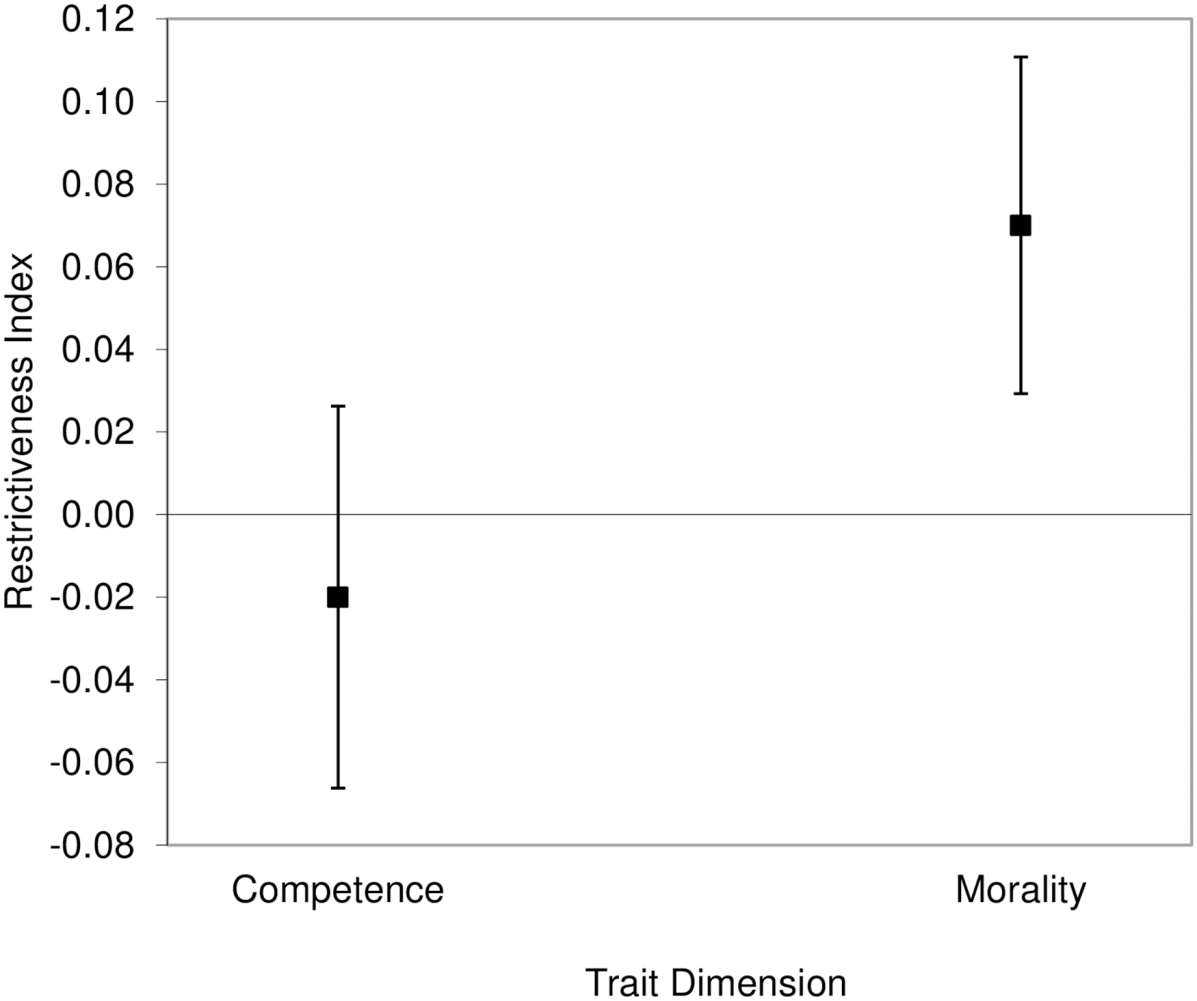
Results of the analysis on the subset of traits in Study 2. The difference between the likelihoods of trait-inconsistent behaviors for the positive and negative poles of traits (restrictiveness index) was significantly positive for morality-related but not competence-related traits. Error bars represent 95% confidence intervals.

Finally, as indicated in the analyses of the larger set of traits, there was a significant difference between the competence *R*_*r*_ index and the morality *R*_*r*_ index (95% CI of the difference [-.14, -.05]), *t*(51) = -3.94, *p* < .001, Cohen’s corrected *d* = .54, indicating higher positive asymmetry of the moral traits versus the competence traits (Fig 4).

## Discussion

The findings of Study 2 largely replicated the pattern of results found in Study 1, even with facilitated question phrasing (with a frequentist format) and a restrictiveness index that incorporated the perceived frequency of behaviors in the population. The participants perceived morality-related traits as positively asymmetric, whereby the positive trait were more likely associated with trait-inconsistent behaviors than the negative traits. For example, the participants assumed that a fair person would more frequently engage in unfair behaviors than an unfair person would perform fair behaviors. Competence-related traits were judged as symmetric. That is, the participants believed that a competent person would be as likely as an incompetent person to perform trait-inconsistent behaviors. These results held both when considering a large set of competence- and morality-related traits and when focusing on the traits that were balanced for valence. Beyond providing converging evidence for the reliability of the results from Study 1, these findings indicate that the information format does not *per se* affect the perceived trait-behavior relations. If anything, the different information formats used in Studies 1–2 could account for the different results from the comparison between the competence and morality restrictiveness indexes. Indeed, in Study 2, the difference in restrictiveness was more pronounced than in Study 1. Competence-related traits were closer to zero (i.e., the value that indicates the symmetry in restrictiveness between the positive and negative trait pole) than in Study 1.

## Study 3

Study 3 addressed the issue of using abstract behavioral categories in the empirical tests of the validity of the implicational schemata (e.g., [17]). The use of trait labels instead of actual behaviors might induce participants to provide biased estimates of behavioral ranges due to the vagueness and variability inherent in trait labels. Therefore, we asked the participants to provide frequency estimates of trait-inconsistent actual behaviors.

## Method

### Participants

Eighty-eight students (77 female, 11 male, *M*_age_ = 24.03, *SD*_age_ = 4.87, range: 19–51 years) volunteered to participate in the study. Eighty-six participants were Italian, one was Egyptian, and one was Romanian. The sample comprised mostly undergraduate students from the faculties of psychology, sociology, economics, mathematics, and physics. One participant was an educator, one was a psychologist, two were freelance professionals, and some were apprentices or student workers.

### Materials and procedure

The study was conducted online by means of the software SurveyMonkey, which is used to create and administer surveys. Participants estimated how often a person with a disposition (e.g., a sincere person) performed a particular trait-inconsistent behavior. Behaviors were drawn from those produced by participants in S5 Text. We selected two trait-inconsistent behaviors for each trait pole based on those most frequently evoked in that study. Therefore, there were four behaviors (two trait-consistent and two trait-inconsistent) for each trait and a total of 24 behaviors. We wanted to ensure that the selected behaviors were perceived by participants as consistent with respect to the hypothesized trait pole. Therefore, we asked participants to estimate the frequency that a person would perform a particular *trait-consistent* behavior. The addition of this manipulation check (which tested the actual association between a given behavior and a trait) doubled the number of questions to 48. Participants responded on an 11-point scale from 0 (*a little*) to 10 (*a lot*). Questions were presented on six screen pages, one for each bipolar trait that was pretested for both relatedness and valence: righteous/unrighteous, sincere/insincere, and fair/unfair for the morality domain and intelligent/stupid, efficient/inefficient, and competent/incompetent for the competence domain. Questions were randomized within each of the six screen pages (i.e., randomization occurred across questions pertaining to the same trait but not across different traits). Furthermore, we randomized the presentation order of the six screen pages. Sample questions for the sincere/insincere trait included: “How often does an insincere person tell the truth?” and “How often does a sincere person omit some information?” (We report the original Italian items together with an English translation in S6 Text).

## Results

### Manipulation check

We first checked that participants perceived the behaviors we selected from the pretest (S5 Text) as actually related to the corresponding traits. Instead of using the scale midpoint (i.e., 5) as a threshold for considering a behavior as associated with the corresponding disposition, we decided to be more conservative and use a higher level in the scale, that is, 6 as a reference point. We performed a series of one-sample *t*-tests to compare the 0–10 estimates that participants provided to manipulation-check questions (e.g., “How often a sincere person tells the truth?”, “How often an insincere person omits some information?”) with 6. We used the Benjamini and Hochberg’s corrected significance levels of *p* = .0479. All of the participants’ estimates (*M*s ≥ 6.85, *SD*s ≤ 2.42), 95% CI of the difference [≥ .45, ≥ 1.26], were significantly higher than the scale point of 6, *t*s(87) ≥ 4.19, *p*s < .001, *d*s ≥ .45. The exception was the question “How often an inefficient person loses herself in details?” for which participants’ mean estimates (*M* = 6.43, *SD* = 2.15), 95% CI of the difference [-.02, .89], were not significantly higher than 6, *t*(87) = 1.89, *p* = .063, *d* = .2. However, the comparison with the scale midpoint (5) was significant, *t*(87) = 6.25, *p* < .001, *d* = .67. Therefore, we concluded that the selected behaviors were perceived by participants as pertaining to the hypothesized correspondent dispositions.

### Analyses of trait-inconsistent behavior frequency

The two frequency estimates of the trait-inconsistent behaviors for each trait pole were significantly positively correlated, Pearson’s *r*s ≥ .21, *N* = 88, *p*s ≤ .049. Therefore, we collapsed over the two judgments that participants provided for each trait pole. We then used (Eq 1) and we computed the difference between the perceived frequency of trait-inconsistent behaviors when the target person has a positive trait and the perceived frequency of trait-inconsistent behaviors when the target person has a negative trait. We compared the combined *R* indexes for competence and morality with zero by using one-sample *t*-tests. We also compared the *R* values of competence- and morality-related traits by means of a paired *t*-test. For each of these three *t*-tests, we used corrected *p*-values of .0333 according to Benjamini and Hochberg’s [37] method. There was symmetry in the frequency of trait-inconsistent behaviors between the positive and negative trait poles for the competence-related traits (*M* = -.11, *SD* = 1.1), 95% CI of the difference [-.34, .13], *t*(87) = -.9, *p* = .370, *d* = .1. As to the morality-related traits, we found a positive asymmetry, that is, a higher frequency of trait-inconsistent behaviors associated with positive trait poles than the reverse (*M* = .28, *SD* = 1.21), 95% CI of the difference [.03, .54], *t*(87) = 2.18, *p* = .032, *d* = .23.

**Fig 5:**
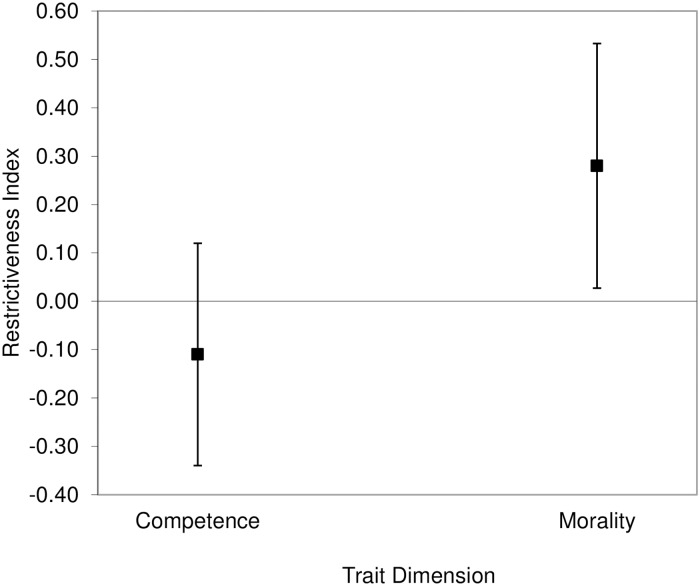
Results of the analysis on the frequency of trait-inconsistent behaviors in Study 3. The difference between the estimated frequencies of trait-inconsistent behaviors for the positive and negative poles of traits (restrictiveness index) was significantly positive for morality- but not competence-related traits. Error bars represent 95% confidence intervals.

There was a significant difference between the collapsed morality-related traits compared with the collapsed competence-related traits (95% CI of the difference [-.72, -.05]), *t*(87) = -2.3, *p* = .024, Cohen’s corrected *d* = .25, indicating a higher positive asymmetry for morality than for competence traits (Fig 5).

S7 Text presents an analysis of the frequency of trait-inconsistent behaviors with a different dependent variable, that is, the “cue-validity index” ([16], p. 692). The findings were consistent with those of the previous analysis showing a positive asymmetry for the morality dimension and symmetry for the competence dimension, although the difference between the morality and competence indexes was not significant.

## Discussion

Study 3 showed that participants tended to associate greater behavioral flexibility with moral rather than immoral target persons when they were presented with concrete categories of behavior. For example, participants perceived it to be more likely that a sincere person would “omit some information” or “cover for somebody” than an insincere person would “tell the truth” or “disclose their feelings to the one they love”. This finding can be interpreted in keeping with the influence of trait favorability on the confirmability and disconfirmability of trait concepts [28]. Indeed, a favorable trait (e.g., sincere) is easier to lose if a person engages in trait-inconsistent behaviors (such as omitting some information) than if she/he is behaviorally restricted. In a similar way, an unfavorable trait (e.g., insincere) is more difficult to lose if a person hardly ever engages in trait-inconsistent behaviors (e.g., telling the truth) than if she/he is behaviorally flexible. With regard to the competence dimension, participants perceived an overall balance between the trait-inconsistent behaviors associated with the positive and negative poles of competence-related traits. For example, they perceived it to be equally likely that an efficient person would “take too much time to complete work” or would “lose themselves in details” and that an inefficient person would “respect their working hours and instructions” or would “commit to completing work”.

## Study 4

Previous research that directly investigated trait-behavior relations was conducted in the US ([16], Experiment 1; [17]) or in the UK ([27]), whereas our studies used samples of Italian participants. Study 4 is cross-cultural and addresses the issue of whether the results of our previous studies generalize across Italian and American participants.

## Method

### Participants

The sample comprised 221 participants (129 female, 92 male, *M*_age_ = 34.05, *SD*_age_ = 12.45, range: 18–68 years). One-hundred and thirteen participants were US citizens (61 female, 52 male, *M*_age_ = 38.44, *SD*_age_ = 12.62, range: 18–68 years), and 108 were Italians (68 female, 40 male, *M*_age_ = 29.45, *SD*_age_ = 10.5, range: 19–68 years).

### Materials and procedure

We devised two online surveys, one for the US sample and one for the Italian one, by means of the software SurveyMonkey. The US sample was recruited and received the link to the SurveyMonkey survey by means of Amazon’s Mechanical Turk. This is a website used by researchers (“requesters”) to post jobs (e.g., surveys) which can be done by participants (“workers”) for pay. The advantage of using Amazon’s Mechanical Turk is that researchers have access to large, diverse samples of participants, rapidly, and at low cost [46,47,48]. We reached participants of the Italian sample by means of social networks such as Facebook.

In either survey, the study was presented as research on “information search in social contexts”. After the introduction page, participants were instructed to estimate how likely a person with a trait would manifest trait-inconsistent behaviors. For example, participants were asked: “How likely do you consider it that a righteous person would behave in an unrighteous fashion?” Then, they were asked the reverse: “How likely do you consider it that an unrighteous person would behave in a righteous fashion?” Participants were asked to click the percentage between 0 (*totally unlikely*) and 100 (*totally likely*). We presented in a single page, in a randomized order, 6 questions related to the competence dimension (i.e., intelligent-stupid, efficient-inefficient, competent-incompetent, stupid-intelligent, inefficient-efficient, incompetent-competent) and 6 questions related to the morality dimension (righteous-unrighteous, sincere-insincere, fair-unfair, unrighteous-righteous, insincere-sincere, unfair-fair) using the trait pairs that were pretested for relatedness and valence.

Finally, the participants were asked to provide their demographic information, that is, gender, age, nationality, profession, and faculty (if student).

## Results

The dependent variable was the difference between the estimates of trait-inconsistent behaviors when the target has a positive trait and the estimates of trait-inconsistent behaviors when the target has a negative trait (see Eq 1).

We computed average scores for competence (Cronbach’s alpha: .75) and morality (Cronbach’s alpha: .67). We then subjected these scores to a 2 (trait dimension: competence vs. morality) × 2 (nationality: American vs. Italian) mixed-design ANOVA, with trait dimension manipulated within participants and nationality as a between-participants variable. We found a significant main effect of trait dimension, *F*(1, 219) = 8.75, *p* = .003, η^2^ = .038, showing that, irrespective of participants’ nationality, estimates were higher for competence (*M* = .07, *SE* = .01), 95% CI [.05, .1], than for morality (*M* = .03, *SE* = .01), 95% CI [.01, .06], 95% CI for difference [.01, .06]. There was also a significant main effect of nationality, *F*(1, 219) = 10, *p* = .002, η^2^ = .040, indicating that the difference scores were overall significantly higher for Italian (*M* = .09, *SE* = .02), 95% CI [.06, .12], than for US participants (*M* = .02, *SE* = .02), 95% CI [-.02, .05], 95% CI for difference [.03, .12]. Crucially to test the hypothesis that our findings hold cross-culturally, nationality did not interact with trait dimension, *F*(1, 219) = .13, *p* = .724, η^2^ = .000.

**Fig 6:**
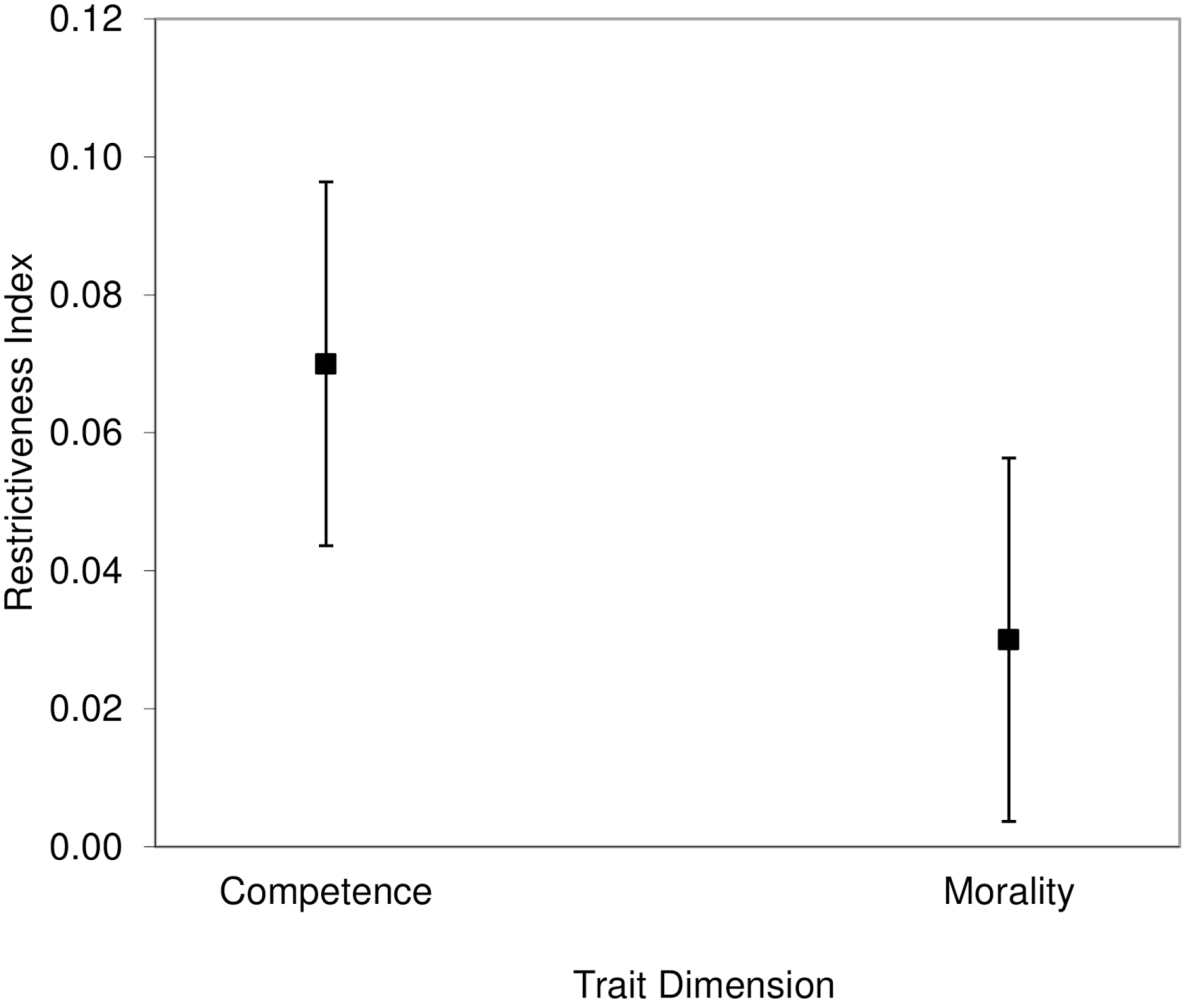
Results of the analysis on the likelihood of trait-inconsistent behaviors in Study 4. The difference between the likelihoods of trait-inconsistent behaviors for the positive and negative poles of traits (restrictiveness index) was significantly positive for both morality-related and competence-related traits. Error bars represent 95% confidence intervals.

Finally, we compared the competence and morality indexes with zero by means of one-sample *t*-tests. Since the nationality by trait dimension interaction was not significant, we analyzed the data across a single sample encompassing both Americans and Italians. The morality index (*M* = .03, *SD* = .2), 95% CI of the difference [.01, .06], was significantly different from zero, *t*(220) = 2.38, *p* = .018, *d* = .16. Also the difference between the competence index (*M* = .07, *SD* = .2), 95% CI of the difference [.04, .1], and zero was significant, *t*(220) = 5.2, *p* < .001, *d* = .35 (Fig 6).

## Discussion

The results of Study 4 showed that the findings of the previous studies hold cross-culturally. Indeed, both American and Italian participants provided the same pattern of likelihoods of trait-inconsistent behaviors for competence and morality. However, the significant main effect of nationality indicates that the perception of positive hierarchical restrictiveness was more pronounced among the Italian participants than among the American participants. In particular, both competence and morality indexes were closer to symmetry for Americans than for Italians. We could interpret this finding as evidence of a cultural effect on implicit assumptions about trait-behavior relations. However, crucially, and in line with the favorability account given by Rothbart and Park [28], both competence and morality indexes were non-negative for both Americans and Italians. In other words, participants assumed that target persons with a positive (either competence or morality although, overall, more so for competence) trait would more likely engage in trait-inconsistent behaviors than target persons with a negative trait would.

## Small-scale meta-analysis

According to “the new statistics” by Cumming [49,50], an additional value of 95% CIs is that they provide information about the replicability of the findings. In the long run, a 95% CI will include, on average, 83% of the means of replication experiments. That is, the probability that a replication of one of our study will give a mean that falls within the particular 95% CI we obtained for that study is, on average, .83 ([49], p. 126).

In our studies, we obtained consistent evidence about the hypothesized positive asymmetry of morality-related traits at moderate levels of both traits and behaviors. In all studies, the 95% CI of the difference between zero and the morality index was made up of positive values and it never included the zero (which is the value that indicates the symmetry of restrictiveness between the two trait poles). Furthermore, the effects of the positive asymmetry along the morality dimension were always at least small to moderate in size (*d* range: .37-.7) according to the threshold proposed by Cohen [51], with the exception of Study 4 (*d* = .16) and one of the dependent variables (the (Eq 1) index) used in Study 3 (*d* = .23).

The relative comparison between morality-related and competence-related trait-behavior relations is not consistent across the studies we conducted. Looking at the 95% CI for the difference between morality and competence, this did not include the zero in Study 2 and when we used the (Eq 1) index in Study 3. In those studies, the direction was toward a higher positive asymmetry for morality than competence. However, the effect was small in Study 3 (Cohen’s corrected *d*: .25). Only in Study 2 the effects were large and moderate (Cohen’s corrected *d*s = .87 and .54). In Study 4, the 95% CI for the difference between morality and competence did not include the zero either, but it indicated a higher positive asymmetry for competence than for morality although the effect was small (Cohen’s corrected *d* = .21). In contrast, the 95% CIs of the difference included the zero, thus indicating no significant differences between competence and morality, in Study 1, in the pretest reported in S5 Text, and in S7 Text when using the “cue-validity index” ([16], p. 692). The effects were tiny (Cohen’s corrected *d*s range: .05-.1) except for the pretest described in S5 Text (*d* = .39).

Cumming [49] recommended the small-scale meta-analysis as a technique that allows researchers to combine the results obtained from different studies that investigate similar questions to increase the precision of the parameter estimates. Therefore, to both increase precision and reconcile inconsistencies across studies about the morality vs. competence comparisons, we meta-analytically combined the results from the effect sizes reported in Studies 1–4 (*N* = 409). We considered the data on the larger sets of traits for Studies 1–2, the findings of Study 4 and we used the results obtained with the (Eq 1) index in Study 3, whereas we did not consider either the results of the pretest reported in S5 Text or the findings obtained with the “cue-validity index” ([16], p. 692) presented in S7 Text.

The meta-analysis showed that the weighted combined *Z*-score for the asymmetry indices between competence and morality was not statistically significant, *Z* = -.62, *p* = .95. Furthermore, the effect size for the asymmetry indices between competence and morality was close to zero, *r* = .19, *d* = -.06. The meta-analysis conducted on both the competence and the morality indices with zero, which represents the perfect symmetry, showed that both competence-related traits, *Z* = 4.23, *p* < .001, and morality-related traits, *Z* = 4.29, *p* < .001, significantly differed from zero.

The effect sizes for both competence, *r* = .07, *d* = .43, and morality, *r* = .33, *d* = .43, were small to medium. In conclusion, both morality- and competence-related traits were asymmetric on the positive pole.

## General discussion

**Fig 7:**
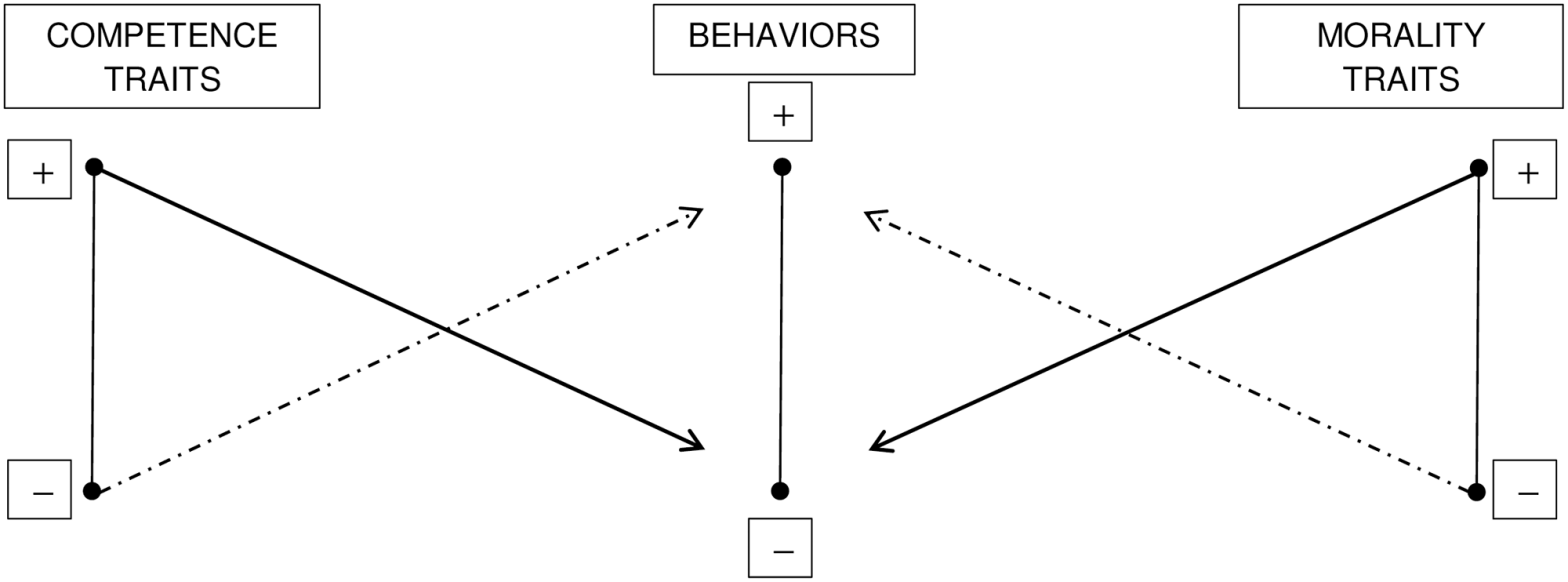
Representation of the hierarchical restrictive schema according to the results of the present research. Positive competence- (left) and morality-related (right) traits (e.g., intelligent and honest) at moderate levels of both traits and behaviors. +, and − indicate positive and negative traits/behaviors, respectively. Solid arrows indicate strong trait-behavior relations, whereas dashed arrows indicate weak trait-behavior relations.

This research provided a unique account of the perceived trait-behavior relations along the morality (versus competence) dimension by focusing on the moderate levels of both traits and behaviors and on the general variability measure (which captures the perceived general frequency of behaviors [17]). We tested the hypothesis of a positive asymmetry in the morality dimension: moral people would be perceived as more likely to act inconsistently than immoral people would. This hypothesis was based on previous theories and findings showing the narrower dispersion associated with immoral traits [29,30,31,32], the lack of negativity effect in information integration when morality-related traits and behaviors are moderate [26], and the favorability effect in trait confirmability and disconfirmability [28]. We used both abstract (Studies 1–2, and Study 4) and concrete (Study 3, see also S5 Text) categories of behaviors and we examined the effect with both Italian and American participants (Study 4). As shown by a small-scale meta-analysis we conducted on data from the four studies, the relation between trait poles and behaviors was positively asymmetric for the morality as well as for the competence dimension. In other words, a person described by a moral trait was thought more likely to emit inconsistent (i.e., immoral) behaviors than an immoral person was thought to engage in inconsistent (i.e., moral) behaviors. We found the same positive asymmetry for competence-related traits. Competent persons were judged more likely to engage in trait-inconsistent behaviors than incompetent target persons (Fig 7).

The similarity of the effect in morality and competence is in keeping with findings from the literature on the confirmability and disconfirmability of traits [28]. Furthermore, historically, there has been an emphasis on cognition in moral functioning which thus might be conceived as affected by competence [52,53]. This view is consistent with classical philosophical work on morality by Aristotle and Kant that pointed out the role of practical reason and wisdom in moral virtue [54,55] (see also [6,12]).

In our analysis, we focused on moderate rather than extreme traits and behaviors as people are more likely to encounter them in everyday life. Furthermore, moderate levels have not been squarely addressed in previous empirical investigations.

### Trait-behavior relations along the morality dimension

In our studies, we found that more behavioral flexibility was associated with target persons with a positive rather than a negative morality-related trait. For example, “covering for somebody” was considered by our participants to be a behavior that could be manifested by sincere persons (although it was perceived as a behavior pertaining to insincerity). Conversely, insincere persons were less likely to be associated with inconsistent behaviors such as “telling the truth” (a behavior associated with sincerity).

The narrower behavioral range that we found for negative trait poles both in the competence and in the morality domain dovetail with range theories of impression formation such as those by Wyer [29] and Birnbaum [30]. Previous studies [29,30] have shown that negative and extreme behaviors have narrower distributions of possible values that can assume than moderate and positive behaviors. Our findings are compatible with this view in that they show that people with negative, moderate traits are perceived as more behaviorally restricted than people with positive, moderate traits regardless of trait content. Our results also dovetail with recent findings on trait attribution and impression updating. An immoral trait is inferred from an immoral behavior more readily than an unsociable trait is inferred from an unsociable behavior, that is, people make immoral assumptions about others [34]. Furthermore, immoral behaviors have more impact on people’s morality evaluations than moral behaviors [56].

Although we did not ask our participants to estimate the number of instances required to confirm or disconfirm that a person possessed a trait, our results might be interpreted coherently also with Rothbart and Park’s [28] findings concerning the influence of trait favorability on the confirmability and disconfirmability of trait concepts. Rothbart and Park found that favorable traits are difficult to acquire but easy to lose, whereas unfavorable traits are easy to acquire and difficult to lose. In our studies, participants perceived that persons with a positive (i.e., favorable) morality trait could be associated with immoral (i.e., unfavorable) behaviors in addition to moral ones. If moral persons are associated also with immoral behaviors, their positive moral disposition might be more easily challenged than if they are perceived as behaviorally restricted. Indeed, the fact that they also engage in immoral behaviors exposes moral persons to more instances that disconfirm their moral disposition than if they engaged almost exclusively in moral behaviors. Along the same lines, in our studies, persons with a negative (i.e., unfavorable) morality trait were associated with more difficulty with trait-inconsistent behaviors than with trait-consistent behaviors. If persons deemed immoral are associated almost exclusively with negative (unfavorable) behaviors, it will be more difficult to disconfirm the unfavorable trait ascription because they are assumed to rarely engage in positive (favorable) moral behaviors.

Our findings concerning the structures of morality traits might be explained by a motivational principle (e.g., [2]). Our participants assumed that moral people (e.g., fair persons) could also behave inconsistently (e.g., unfairly), whereas immoral individuals (e.g., unfair persons) were assumed to hardly ever behave inconsistently (e.g., in a fair way). These implicit assumptions might be the result of the need to avoid the aversive consequences of immoral behaviors. An emphasis on potentially negative consequences in the interpersonal context is in keeping with an over-protective strategy (e.g., [2,57]), whereby people prefer to incur false positive rather than omission errors (see also [33]). Therefore, individuals prefer to hold the preconception that other people might generally behave less morally than a strict definition of their disposition would suggest, whereas the immoral disposition is considered to be stable.

### Inconsistencies in the literature

There are methodological differences that could account for the discrepancies between our findings and results of classic work on the same topic (e.g., [27], Study 3; [16], Experiment 1). Tausch et al.’s [27] Study 3 used a “diagnosticity” measure similar to the one we used and they focused on moderate trait-behavior relations as we did. However, the authors of that study considered the broader dimension of warmth rather than only morality-related traits. Furthermore, they used only abstract behavioral categories to test the likelihood of trait-inconsistent behaviors (see [27], p. 550), as did Reeder et al. [17]. Skowronski and Carlston ([16], Experiment 1) used concrete behaviors, but considered only one moderate trait (i.e., honest/dishonest) on the morality dimension (and one moderate trait, that is, intelligent/stupid on the competence dimension). Furthermore, they examined five different behavioral levels (i.e., “extremely honest”, “moderately honest”, “honesty neutral”, “moderately dishonest”, and “extremely dishonest”). Similarly to Tausch et al., in their analysis collapsing over the extremity of behaviors, they found the negative asymmetry in morality and positive asymmetry in competence that we should expect for *extreme* levels of both traits and behaviors and for the *moderate* trait-*extreme* behavior relations [17].

In our studies, we focused on a systematic analysis of trait-behavior relations at the moderate levels of both traits and behaviors. Our studies did not examine whether trait-behavior relations differed as a function of the level of the extremity of either the disposition or the behavior. It is likely that very extreme immoral behaviors, such as murders or terrorist acts (e.g., “placing razor blades in children’s Halloween apples”, [18], p. 69), are not perceived as belonging to the behavioral repertoire of an even moderately moral person (see Fig 1 and [17]). Skowronski and Carlston ([16], Experiment 1) and Tausch et al. ([27], Study 3) have not systematically examined the distinction between moderate and extreme traits and behaviors. Hence, a question for future research is whether and how trait-behavior relations vary as a function of the different levels of extremity of either a trait or a behavior along the morality and competence dimensions using also the potential variability and the intended variability measures, thus further deepening Reeder et al.’s analysis [17]. Future research should also establish whether the different results in our studies and in the study by Skowronski and Carlston ([16], Experiment 1) are due to the influence of different levels of morality-related behaviors on people’s judgments or to a peculiarity of the honest/dishonest trait. At the inferential level of the trait-attribution process, it has been noted that morality traits such as honest/dishonest might entail a different implicational schema than morality traits such as charitable/uncharitable (e.g., [12]).

In addition, in our studies, we did not consider either dispositional factors (e.g., a specific target’s ability to perform a behavior, which is captured by the potential variability measure [17]) or situational factors (e.g., social desirability, which affects the attempts to emit certain behaviors and it is captured by the intended variability measure [17]) that might affect trait-behavior relations.

For all these reasons, the overall wider range of behaviors associated with moral dispositions relative to immoral dispositions that we found is not necessarily in contrast with previous findings concerning morality attributions that showed, for example, that morality attributions were less affected by situational demands when immoral rather than moral behaviors were observed (e.g., [24]). In other words, our results are not necessarily at odds with the negativity biases characteristic of the moral domain (e.g., [58]). In contrast, they show how negative assumptions about someone’s morality are relevant not only in trait attributions [34], but also where trait-behavior relations are concerned. People might hold the implicit assumption that individuals with a moral disposition might also engage in behaviors that are inconsistent with their disposition, as the results of our studies suggest. However, when forming an impression based on an observed immoral behavior (e.g., [35], Experiment 2), people might still tend to weigh this non-correspondence heavily due to the negativity bias (e.g., [14,59]) or the adjustments made to consider social and situational demands (e.g.,[20,21,60,61]). This is even more likely if the trait attribution is based on extreme behavior instances rather than the moderate behaviors we examined in our studies. Indeed, Wojciszke and colleagues [26] found that the negativity bias in the morality domain disappeared when participants made trait inferences and evaluations of a target described by moderate rather than extreme morality-related behaviors. Within this framework, people’s final attribution based on immoral behaviors could still be correspondent and less subject to the influence of social pressure, situational demands, or contextual factors (e.g., costs or rewards) than their attributions based on moral instances [20,24]. Furthermore, they will readily reach a negative conclusion about someone’s morality based on an immoral behavior [34].

### Future directions

Given the limited research conducted on moderate trait-behavior relations, our research might provide scholars with traits and behaviors that could be used in future studies. In our studies, we used different indexes to quantify the restrictiveness of a personality trait (see Eqs (1), (5), and, in S7 Text, Equation (7)). These indexes represent different, but converging ways of characterizing the perceived likelihood of trait-inconsistent behaviors at the two poles of a trait. Future studies might investigate whether and to what extent different degrees of restrictiveness elicit different expectations (e.g., [42,62]), search strategies (e.g., [45,63,64,65]), or final trait inferences.

A further extension of this research could address the issue of the neurophysiological correlates of the perceived positive asymmetry in morality trait-behavior relations. Prior work has shown that semantic anomalies elicited a N400 component in event-related brain potential (ERPs, e.g., [66,67]; for a review, see [68]). By recording ERPs from participants who read trait-consistent and trait-inconsistent behaviors referring to a moral/immoral target person, it would be possible to see whether N400 is elicited or, in line with our results, whether semantic anomalies are not perceived by participants when reading about immoral behaviors associated with moral persons.

Our findings can be further specified in light of research on stereotyping. Trope’s [69] and Reeder’s [20] attribution models assume that the initial identification of a target’s behavior and of a target’s trait is influenced, among other factors, by prior information such as the actor’s social group membership and stereotypes about the target person. Future studies could investigate whether prior information, in particular stereotypes, might also influence the implicit assumptions about trait-behavior relations that guide the correction stage [20]. Reeder envisaged this possibility in the discussion of his model of trait inference by arguing that variables that influence the initial dispositional characterization of the actor could interact with trait-behavior relations (see [20], p. 592). Trait stereotypicality and social group membership might shape perceived trait-behavior relations. More precisely, we might expect a stronger positive asymmetry for traits that are stereotypical of an outgroup (e.g., “intelligent” and “religious”, traits that are considered stereotypic of Jews by Italians [70]). Along the lines of Biernat and Ma’s study [39] on trait confirmability, future studies could thus address how stereotyping affects trait-behavior relations. In line with this approach that looks at the impact of trait-behavior relations on intergroup relations, Duran, Renfro, Waller, and Trafimow [71] found that a hierarchically restrictive schema can characterize not only trait-behavior relations, but also behavior-group membership relations. In particular, they found that sexual orientation is a hierarchically restrictive trait.

The results of our research cast light on a neglected area of the attribution research, but, besides their theoretical relevance, they also bear potential implications for contexts in which personality traits and their perception can influence decision making. For instance, holding a negative assumption about a defendant’s morality, such as a focal hypothesis of guilt, can contribute, if no corrections take place, to judgmental biases in legal contexts. As for the assumptions underlying the competence dimension, concerns about someone’s ability within work and organizational contexts might lead to conservatism in hiring decisions, negotiations, and performance appraisals.

## Conclusions

The present research extended previous work on one of the determinants of trait attribution, namely, trait-behavior relations. In particular, we systematically examined the moderate levels of the perceived behavioral range associated with the positive and negative poles of a trait continuum by using different information formats (probability vs. frequency), categories of behaviors (abstract vs. concrete), and participants’ nationality (Italians vs. Americans). The results indicated that trait-behavior relations for morality traits are positively asymmetric, with the positive pole of a trait being perceived as more behaviorally unrestricted than the negative one. Competence-related traits were also perceived as positively asymmetric, with target persons at the positive pole of the trait continuum being seen as more behaviorally flexible than those at the negative pole. These findings shed light on some inconsistencies in the literature (e.g., [27]). They also cohere with previous results from range theories (e.g., [29,30,31,32]), information integrations studies [26], on the influence of trait favorability on the confirmability and disconfirmability of trait concepts [28], on impression updating [56] and on trait assumptions [34].

## Supporting information

S1 Table Pretest on Trait-Domain Relatedness. (DOCX)

S1 Text General variability as a measure of trait-behavior relations. (DOCX)

S2 Text Preliminary study. (DOCX)

S3 Text Trait valence pretest. (DOCX)

S4 Text Participants’ prior probability estimates of traits and derived probabilities of occurrence of behaviors. (DOCX)

S5 Text Pretest on concrete trait-inconsistent behaviors. (DOCX)

S6 Text Original version in Italian and the English translation of the 48 items used in Study 3. (DOCX)

S7 Text Analyses of trait-inconsistent behavior frequency in Study 3 with the cue-validity index. (DOCX)
