# Supplementary material for: You are fair, but I expect you to also behave unfairly: Positive asymmetry in trait-behavior relations for moderate morality information
Source: PLoS One. 2017 Jul 11;12(7):e0180686. doi: 10.1371/journal.pone.0180686 (PMC5507453; doi:10.1371/journal.pone.0180686)
Supplement: S1 Table — (DOCX) [file pone.0180686.s001.docx]

**S1 Table**

**S1 Table. Pretest on Trait-Domain Relatedness**. Results of the One-Sample T-Tests Comparing Participants’ Ratings with the Scale Midpoint (i.e., 4 ) for Twenty-Seven Traits. We Selected the First Twenty-Three Traits

| Trait | Domain | Mean (*SD*) | 95% CI | *t*-value (df) | *p*-value | Cohen’s *d* |
| --- | --- | --- | --- | --- | --- | --- |
| Intelligent/stupid | Morality | 3.69 (1.99) | [3.26, 4.12] | -1.41 (84) | .161 | .15 |
|  | Competence | 4.64 (2.06) | [4.19, 5.09] | 2.86 (83) | .005 | .31 |
| Skillful/unskillful | Morality | 3.1 (2.1) | [2.64, 3.56] | -3.95 (83) | < .001 | .43 |
|  | Competence | 5.02 (2.02) | [4.58, 5.46] | 4.61 (82) | < .001 | .51 |
| Efficient/inefficient | Morality | 3.67 (2.01) | [3.24, 4.1] | -1.51 (84) | .134 | .16 |
|  | Competence | 4.96 (1.87) | [4.56, 5.36] | 4.76 (84) | < .001 | .52 |
| Competence/incompetence | Morality | 3.36 (2.2) | [2.88, 3.84] | -2.65 (82) | .010 | .29 |
|  | Competence | 5.54 (2.02) | [5.1, 5.98] | 7.03 (84) | < .001 | .76 |
| Able/unable | Morality | 3.42 (2.03) | [2.98, 3.86] | -2.63 (83) | .010 | .29 |
|  | Competence | 5.01 (2.03) | [4.57, 5.45] | 4.58 (83) | < .001 | .5 |
| Expert/inexpert | Morality | 3.27 (2.16) | [2.8, 3.74] | -3.12 (84) | .002 | .34 |
|  | Competence | 4.92 (2.11) | [4.46, 5.38] | 3.98 (83) | < .001 | .43 |
| Prepared/unprepared | Morality | 3.72 (2.16) | [3.25, 4.19] | -1.2 (84) | .232 | .13 |
|  | Competence | 5.19 (1.93) | [4.77, 5.61] | 5.68 (84) | < .001 | .62 |
| Valuable/invaluable | Morality | 3.8 (1.89) | [3.39, 4.21] | -.98 (83) | .330 | .11 |
|  | Competence | 4.61 (1.82) | [4.21, 5.01] | 3.08 (82) | .003 | .34 |
| Pure/impure | Morality | 4.51 (2.17) | [4.03, 4.99] | 2.14 (81) | .035 | .24 |
|  | Competence | 3.25 (2.06) | [2.8, 3.7] | -3.3 (82) | .001 | .36 |
| Honest/dishonest | Morality | 5.46 (1.97) | [5.03, 5.89] | 6.83 (83) | < .001 | .74 |
|  | Competence | 3.6 (2.23) | [3.12, 4.08] | -1.66 (84) | .101 | .18 |
| Altruistic/selfish | Morality | 5.18 (1.8) | [4.79, 5.57] | 5.99 (83) | < .001 | .65 |
|  | Competence | 3.38 (2.04) | [2.94, 3.82] | -2.82 (84) | .006 | .31 |
| Righteous/unrighteous | Morality | 4.39 (1.81) | [4, 4.78] | 1.98 (84) | .051 | .21 |
|  | Competence | 3.64 (1.88) | [3.23, 4.05] | -1.79 (84) | .077 | .19 |
| Loyal/disloyal | Morality | 5.35 (2.05) | [4.91, 5.79] | 6.1 (84) | < .001 | .66 |
|  | Competence | 3.85 (2.19) | [3.38, 4.32] | -.64 (84) | .521 | .07 |
| Faithful/unfaithful | Morality | 5.24 (2.17) | [4.77, 5.71] | 5.22 (82) | < .001 | .57 |
|  | Competence | 3.47 (2.17) | [3, 3.94] | -2.22 (82) | .029 | .24 |
| Sincere/insincere | Morality | 5.06 (2.15) | [4.6, 5.52] | 4.55 (84) | < .001 | .49 |
|  | Competence | 3.6 (2.14) | [3.14, 4.06] | -1.72 (84) | .089 | .19 |
| Respectful/disrespectful | Morality | 5.08 (1.9) | [4.67, 5.49] | 5.22 (83) | < .001 | .57 |
|  | Competence | 4.39 (2.08) | [3.94, 4.84] | 1.72 (84) | .089 | .19 |
| Tolerant/intolerant | Morality | 4.84 (1.77) | [4.45, 5.23] | 4.34 (82) | < .001 | .48 |
|  | Competence | 4.04 (1.87) | [3.63, 4.45] | .18 (83) | .861 | .02 |
| Correct/incorrect | Morality | 5.25 (1.8) | [4.86, 5.64] | 6.39 (84) | < .001 | .69 |
|  | Competence | 4.04 (2.02) | [3.6, 4.48] | .16 (84) | .872 | .02 |
| Direct/indirect | Morality | 4.6 (1.92) | [4.18, 5.02] | 2.84 (83) | .006 | .31 |
|  | Competence | 3.59 (1.89) | [3.18, 4] | -1.98 (82) | .051 | .22 |
| Frank/evasive | Morality | 4.55 (1.78) | [4.17, 4.93] | 2.86 (84) | .005 | .31 |
|  | Competence | 3.54 (1.73) | [3.16, 3.92] | -2.42 (82) | .018 | .27 |
| Principled/unprincipled | Morality | 4.51 (1.96) | [4.08, 4.94] | 2.36 (81) | .021 | .26 |
|  | Competence | 3.48 (1.77) | [3.09, 3.87] | -2.67 (82) | .009 | .29 |
| Fair/unfair | Morality | 4.71 (1.79) | [4.32, 5.1] | 3.65 (84) | < .001 | .40 |
|  | Competence | 3.89 (1.9) | [3.48, 4.3] | -.51 (84) | .608 | .06 |
| Having integrity/corrupt | Morality | 4.99 (1.91) | [4.58, 5.4] | 4.77 (84) | < .001 | .52 |
|  | Competence | 3.8 (2.11) | [3.34, 4.26] | -.88 (82) | .379 | .1 |
| Obedient/disobedient | Morality | 4.15 (1.83) | [3.76, 4.54] | .77 (84) | .443 | .08 |
|  | Competence | 3.32 (1.76) | [2.94, 3.7] | -3.54 (83) | .001 | .39 |
| Reliable/unreliable | Morality | 4.95 (1.62) | [4.6, 5.3] | 5.41 (84) | < .001 | .59 |
|  | Competence | 4.49 (2.04) | [4.05, 4.93] | 2.19 (83) | .031 | .24 |
| Discerning/undiscerning | Morality | 3.66 (1.82) | [3.27, 4.05] | -1.73 (84) | .088 | .19 |
|  | Competence | 4.31 (1.81) | [3.92, 4.7] | 1.56 (84) | .124 | .17 |
| Creative/uncreative | Morality | 3.22 (1.8) | [2.83, 3.61] | -3.97 (84) | < .001 | .43 |
|  | Competence | 4.02 (1.96) | [3.6, 4.44] | .11 (84) | .912 | .01 |
