# Supplementary material for: You are fair, but I expect you to also behave unfairly: Positive asymmetry in trait-behavior relations for moderate morality information
Source: PLoS One. 2017 Jul 11;12(7):e0180686. doi: 10.1371/journal.pone.0180686 (PMC5507453; doi:10.1371/journal.pone.0180686)
Supplement: S1 Text — (DOCX) [file pone.0180686.s002.docx]

**S1 General Variability as a Measure of Trait-Behavior Relations**

The measure called “general variability” by Reeder, Henderson, and Sullivan (1982) closely mimics the measures used in subsequent studies that made key contributions to the field, namely those by Skowronski and Carlston (1987) and Tausch, Kenworthy, and Hewstone (2007). Skowronski and Carlston (1987) specifically addressed trait-behavior relations in their Experiment 1. They asked participants questions in the form: “Would a (trait) person ever (behavior)?” (Skowronski & Carlston, 1987, p. 691). For example: “Would an honest (dishonest) person ever search for the owner of a lost package?” (Skowronski & Carlston, 1987, pp. 691-692). Their instructions read: “(…) We are interested in your views of the frequency with which people with various personality traits perform both consistent and inconsistent behaviors” (Skowronski & Carlston, 1987, p. 692). Furthermore, “ratings were made on a 9-point scale that ranged from *extremely unlikely* to *extremely likely to perform the behavior*, with *moderately likely to perform the behavior* at the midpoint” (Skowronski & Carlston, 1987, p. 692).

The operationalization in terms of likelihood of trait-inconsistent behaviors has also been used by Tausch et al. (2007, Study 3). The authors did not report the precise phrasing of their measure, but they described it as follows: “how likely it is that someone who possesses the given trait shows trait-inconsistent behaviors (*diagnosticity*)” (Tausch et al., 2007, p. 550).

In sum, both Skowronski and Carlston’s (1987) and Tausch et al.’s (2007) instructions tap into the same definition Reeder et al. (1982) used for the “general variability” measure: “ratings of the frequency with which persons are thought to actually engage in behaviors” (Reeder et al., 1982, p. 359).

Beyond allowing us to compare our findings with previous research in the field, there are also theoretical considerations that justify our focus on the “general variability” measure used by Reeder et al. (1982). The measures the authors called “intended variability” and “potential variability” do not isolate trait-behavior relations. Indeed, questions about the perceived attempts to perform behaviors (“intended variability”, Reeder et al., 1982) emphasize the influence of situational demands (“if a large reward were available for doing so”, Reeder et al., 1982, p. 361) on the perceived ranges of behaviors associated with dispositions. The underlying rationale is that considerations of the social desirability of traits and behaviors could influence trait-behavior relations (Reeder et al., 1982). Although context is a key factor in the attribution process (e.g., Jones & Davis, 1965; Kelley, 1973), we wanted to isolate trait-behavior relations from the influence of situational demands.

Questions about the perceived role-taking ability (“potential variability”, Reeder et al., 1982) asked about an inherently competence-related dimension, as in the study by Reeder, Messick, and Van Avermaet (1977). For example, asking about the extent to which a person who is very honest “can adequately portray” (Reeder et al., 1982, p. 361) a person who is very dishonest captures the ability component required by role taking rather than whether honest and dishonest persons have equal behavioral flexibility.

Accordingly, we focused on questions about the perceived general frequency of behaviors (“general variability”, Reeder et al., 1982), that is, perceptions of what people actually do, because their phrasing isolates trait-behavior relations from their determinants such as social desirability and ability (Reeder et al., 1982).
