# Supplementary material for: You are fair, but I expect you to also behave unfairly: Positive asymmetry in trait-behavior relations for moderate morality information
Source: PLoS One. 2017 Jul 11;12(7):e0180686. doi: 10.1371/journal.pone.0180686 (PMC5507453; doi:10.1371/journal.pone.0180686)
Supplement: S2 Text — (DOCX) [file pone.0180686.s003.docx]

**S2 Preliminary Study**

We presented 85 participants (51 females, 34 males, 81 Italians, two Romanians, one Brazilian, 83 undergraduate students, one attorney, one participant did not provide her/his nationality and profession, *M*_age_ = 21.38, *SD*_age_ = 2.64, range 19-35 years) with a set of 27 traits which we thought that could be related either to the competence or to the morality dimension. We then asked participants to rate each of these traits in terms of their relatedness to both the competence/incompetence and morality/immorality dimension on a 7-point scale ranging from 1 (*little*) and 7 (*a lot*). We varied the following variables between-participants: Question order (morality/immorality rating first, competence/incompetence rating then, and the reverse order), trait order (the original order and the reverse one), and trait valence (positive, e.g., “intelligent” versus negative, e.g., “stupid”). Therefore, there were eight versions of the questionnaire. A sample question was: “Intelligence is a characteristic that refers to the domain of…”, then participants were presented with the two 7-point scales, one labeled Morality/Immorality and the other one labeled Competence/Incompetence.

We conducted 54 one-sample *t*-tests comparing participants’ ratings with the midpoint of the scale (i.e., 4). We used adjusted alpha levels of .0340 per test according to the Benjamini and Hochber’s (1995) correction. We selected the traits that participants rated significantly higher than the scale midpoint for the related dimension and significantly below or not significantly different from the scale midpoint for the unrelated dimension. For example, we considered “intelligent/stupid” as a competence-related trait because participants rated this characteristic significantly higher than the midpoint of the competence/incompetence relatedness scale, and not significantly different from the midpoint of the morality/immorality relatedness scale. The comparisons against the morality scale’s midpoint were only marginally significant for two traits, namely righteous/unrighteous and pure/impure. However, we included them in the morality-related trait set because the Cohen’s *d*s were higher than .2, which is the threshold proposed by Cohen (1988) for a small effect, and they were not significantly related to competence. S1 Table shows the results of the analyses for the twenty-three traits that we eventually selected as well as for the four traits (i.e., obedient/disobedient, reliable/unreliable, discerning/undiscerning, creative/uncreative) that we excluded.
