# Supplementary material for: You are fair, but I expect you to also behave unfairly: Positive asymmetry in trait-behavior relations for moderate morality information
Source: PLoS One. 2017 Jul 11;12(7):e0180686. doi: 10.1371/journal.pone.0180686 (PMC5507453; doi:10.1371/journal.pone.0180686)
Supplement: S3 Text — (DOCX) [file pone.0180686.s004.docx]

**S3 Trait Valence Pretest**

The same participants from the trait-relatedness pretest rated also the 23 pretested traits on a 7-point scale ranging from -3 (*very negative*) to 3 (*very positive*), with a midpoint of 0 (*neutral*). A sample question was: “Intelligence is a characteristic...” (see S2 for details on the materials and procedure used). For each of the 10 following *t*-tests we used adjusted significance levels of .0400 based on Benjamini and Hochberg’s (1995) correction. Based on the participants’ valence ratings, we selected the competence- and morality-related traits that were comparable in terms of valence. We found that only the following two subsets consisting of three traits each were not significantly different in terms of valence according to paired *t*-tests and thus were sufficiently balanced: intelligent/stupid, efficient/inefficient, and competent/incompetent for the competence domain and righteous/unrighteous, sincere/insincere, and fair/unfair for the morality domain. In particular, the participants’ valence ratings for the positively valenced poles of the three morality traits (righteous, sincere, and fair (*M* = 1.98, *SD* = .66)) did not differ significantly from their ratings of the positively valenced poles of the three competence traits (intelligent, efficient, and competent (*M* = 2.11, *SD* = .61)), 95% CI of the difference [-.36, .1], *t*(42) = -1.16, *p* = .251, Cohen’s corrected *d* = .17. Similarly, there was no significant difference in the participants’ valence ratings between the three negatively valenced morality traits (unrighteous, insincere, and unfair (*M* = -1.45, *SD* = .75)) and the three negatively valenced competence traits (stupid, inefficient, and incompetent (*M* = -1.28, *SD* = .74)), 95% CI of the difference [-.45, .11], *t*(41) = -1.22, *p* = .229, Cohen’s corrected *d* = .19 . In light of these results, we concluded that the selected sets of three morality traits and three competence traits were sufficiently balanced and comparable in terms of valence to avoid any possible valence-related confounders in the competence versus morality comparison.

We then checked that the selected trait subsets were not asymmetric within each domain (in the moral versus immoral and competence versus incompetence dimensions). For each domain, we compared both the positive traits and the negative traits with the scale midpoint (i.e., 0) using one-sample *t*-tests. The three negative moral traits were significantly different from the scale midpoint, 95% CI of the difference [-1.68, -1.22], *t*(41) = -12.47, *p* < .001, *d* =1.92. Similarly, the three positive moral traits were significantly different from the scale midpoint, 95% CI of the difference [1.78, 2.18], *t*(42) = 19.75, *p* < .001, *d* = 3.01. We found the same pattern within the competence domain. The three negative competence traits differed significantly from the scale midpoint, 95% CI of the difference [-1.51, -1.05], *t*(41) = -11.2, *p* < .001, *d* = 1.73. Finally, the three positive competence traits were significantly higher than the scale midpoint, 95% CI of the difference [1.92, 2.3], *t*(42) = 22.77, *p* < .001, *d* = 3.47. In light of these results, we considered the selected traits sufficiently balanced for valence within the respective domains.

Although the adverb “very” was not added to the trait adjectives we presented to participants, we wanted to check that the subsets of competence-related and morality-related traits were not perceived as extreme, but moderate. A significant difference from the scale endpoints, that is, -3 (*very negative*) and 3 (*very positive*), would provide evidence that the traits we used were moderately moral/immoral or competent/incompetent. The three negative moral traits were significantly different from the negative scale endpoint (i.e., -3), 95% CI of the difference [1.32, 1.79], *t*(41) = 13.35, *p* < .001, *d* = 2.06. The three positive moral traits were also significantly different from the positive scale endpoint (i.e., 3), 95% CI of the difference [-1.23, -.82], *t*(42) = -10.22, *p* < .001, *d* = 1.56. In a similar way, the three negative competent traits were significantly different from the negative scale endpoint, 95% CI of the difference [1.49, 1.95], *t*(41) = 15.09, *p* < .001, *d* = 2.33. Finally, the three positive competence traits were also significantly different from the positive scale endpoint, 95% CI of the difference [-1.08, -.7], *t*(42) = -9.62, *p* < .001, *d* = 1.47. All the comparisons with the scale endpoints had large effect sizes, thus providing evidence that the traits we selected were perceived as moderate.
