# Supplementary material for: You are fair, but I expect you to also behave unfairly: Positive asymmetry in trait-behavior relations for moderate morality information
Source: PLoS One. 2017 Jul 11;12(7):e0180686. doi: 10.1371/journal.pone.0180686 (PMC5507453; doi:10.1371/journal.pone.0180686)
Supplement: S4 Text — (DOCX) [file pone.0180686.s005.docx]

**S4 Participants’ Prior Probability Estimates of Traits and Derived Probabilities of Occurrence of Behaviors**

Participants in Study 2 provided estimates of the number of persons out of 10 possessing a trait, that is, $p\left( H \right)$. From those estimates we derived the probabilities of occurrence of behaviors, that is,$p\left( D \right)$. We present the analyses on both participants’ estimates and the derived probabilities.

**Estimates of the Prior Probabilities of the Traits**

We averaged the estimates of trait prevalence among people, that is, $p\left( H \right)$, for the eight competence-related traits (Cronbach’s alpha = .89) and the 15 morality-related traits (Cronbach’s alpha = .91). We then compared each of these composite scores against the chance level (*p* = .5) that would indicate a random prevalence of the traits in the population by means of two one-sample *t*-tests. Finally, we compared the competence and the morality scores to each other by means of a paired *t*-test. For each of these three tests, we used adjusted alpha levels of .0333 following the Benjamini-Hochberg’s (1995) correction for multiple testing. The comparison between the competence score (*M* = .52, *SD* = .14), 95% CI of the difference [-.02, .06], and .5 was not significant, *t*(51) = 1.03, *p* = .310, *d* = .14. In contrast, the morality score (*M* = .41, *SD* = .11), 95% CI of the difference [-.12, -.05] was significantly lower than 0.5, *t*(51) = -5.43, *p* < .001, *d* = .75. Finally, the morality score was significantly lower than the competence score, 95% CI of the difference [.07, .14], *t*(51) = 6.62, *p* < .001, Cohen’s corrected *d* = .98.

We conducted the same analyses on the subsets of traits that were balanced for valence using the same adjusted alpha levels to correct for multiple testing. The estimates for the three positive competence-related traits (i.e., intelligent, efficient, competent, Cronbach’s alpha = .79) and those for the three morality-related traits (i.e., righteous, sincere, fair, Cronbach’s alpha = .77) were averaged in two composite scores. The results were consistent with those of the analyses on the large set of traits. The competence score (*M* = .51, *SD* = .15), 95% CI of the difference [-.04, .05], was not significantly different from .5, *t*(51) = .34, *p* = .738, *d* = .05. In contrast, the morality score (*M* = .42, *SD* = .13), 95% CI of the difference [-.11, -.04] was significantly lower than .5, *t*(51) = -4.32, *p* < .001, *d* = .6. Finally, the morality score was significantly lower than the competence score, 95% CI of the difference [.05, .12], *t*(51) = 4.42, *p* < .001, Cohen’s corrected *d* = .66.

**Derived Probabilities of Occurrence of Behaviors**

Similarly to the analyses on the prior probability estimates, we examined the derived probabilities of occurrence of behaviors, that is,$p\left( D \right)$, for the eight competence-related traits (Cronbach’s alpha = .9) and the 15 morality-related traits (Cronbach’s alpha = .93). We collapsed the computed $ps\left( D \right)$ across traits to create two composite scores for competence and morality, respectively. We then compared these scores against the chance level (*p* = .5) that would indicate a random probability of occurrence of the behaviors by means of two one-sample *t*-tests. Finally, we compared the competence and the morality scores to each other by means of a paired *t*-test. For each of these three tests, we used adjusted alpha levels of .0333 following the Benjamini-Hochberg’s (1995) correction for multiple testing. The competence score (*M* = .49, *SD* = .12), 95% CI of the difference [-.04, .02], was not significantly different from and .5, *t*(51) = -.53, *p* = .595, *d* = .07. In contrast, the morality score (*M* = .44, *SD* = .11), 95% CI of the difference [-.09, -.03] was significantly lower than .5, *t*(51) = -4.06, *p* < .001, *d* = .56. Finally, the morality score was significantly lower than the competence score, 95% CI of the difference [.03, .08], *t*(51) = 4.09, *p* < .001, Cohen’s corrected *d* = .54.

We conducted the same analyses, using the same adjusted alpha levels, on the subsets of three competence-related traits (Cronbach’s alpha = .72) and three morality-related traits (Cronbach’s alpha = .76) balanced for valence. The results were in keeping with those found in the previous analyses and they showed that the difference between the composite competence score (*M* = .48, *SD* = .12), 95% CI of the difference [-.05, .02], and .5 was not significant, *t*(51) = -.9, *p* = .373, *d* = .12. In contrast, the composite morality score (*M* = .45, *SD* = .11), 95% CI of the difference [-.08, -.02] was significantly lower than .5, *t*(51) = -3.33, *p* = .002, *d* = .46. Finally, the morality score was significantly lower than the competence score, 95% CI of the difference [.01, .07], *t*(51) = 2.61, *p* = .012, Cohen’s corrected *d* = .3.
