# Supplementary material for: You are fair, but I expect you to also behave unfairly: Positive asymmetry in trait-behavior relations for moderate morality information
Source: PLoS One. 2017 Jul 11;12(7):e0180686. doi: 10.1371/journal.pone.0180686 (PMC5507453; doi:10.1371/journal.pone.0180686)
Supplement: S5 Text — (DOCX) [file pone.0180686.s006.docx]

**S5 Pretest on Concrete Trait-Inconsistent Behaviors**

We asked participants to generate a series of behaviors that referred to the two ends of each trait that we pretested for both relatedness and valence. This pretest provided us with a set of concrete behavioral categories that we could use in Study 3. In doing so, we also tested the hypothesis that the symmetric or asymmetric trait-behavior relations would be reflected in a symmetric or asymmetric number of behaviors perceived to be related to the dispositions. In other words, we expected that participants would produce a higher number of behaviors related to the unrestricted end of a trait continuum than to the restricted trait endpoint. This measure based on the *amount* of generated behaviors could provide converging evidence, together with the measure based on the *likelihood* of trait-inconsistent behaviors, for the hypothesized positive asymmetry in the morality and competence domains.

**Method**

**Participants**

Twenty Italian undergraduate students (11 female, 9 male, *M*_age_ = 23.20, *SD*_age_ = 2.44, range: 20-31 years) participated in the study.

**Materials and Procedure**

We conducted this study on computers by means of the Inquisit 3.0.6.0 software, which is used to design and administer psychological questionnaires and experiments. The participants came to the lab and read the instructions for the study on the screen. Before and after the study, the participants could ask for clarification from the experimenter who assisted in the lab. The participants were asked to “list a series of behaviors that can be performed by individuals with a particular personality trait”. Specifically, they were provided with the following example of the question type they would receive: “List a series of extroverted behaviors that, according to you, might also be performed by an introvert”. The instructions told the participants to list as many behaviors as possible for each personality trait. On the subsequent screens, the participants were required to provide their gender, age, and nationality, and then they were presented with behavior-production questions, one for each screen. Questions were presented by the software randomly without replacement. The trait for which participants had to produce trait-inconsistent behaviors were the six traits pretested for valence: three morality-related traits (i.e., righteous/unrighteous, sincere/insincere, and fair/unfair) and three competence-related traits (i.e., intelligent/stupid, efficient/inefficient, and competent/incompetent). There were 12 questions because we asked participants to generate behaviors that referred to both endpoints for each trait. For example, behaviors related to the efficient/inefficient disposition were elicited by means of the following questions: “List a series of efficient behaviors that, according to you, might also be performed by an inefficient person” and on a different screen, “list a series of inefficient behaviors that, according to you, might also be performed by an efficient person”. This type of instructions encouraged participants to avoid producing instances of extreme trait-inconsistent behaviors. The open-ended questions had a maximum of 100,000 characters for responses. The participants could skip questions, but they could not return to previous questions. There were no time constraints. Upon completion of the task, the participants were thanked and released.

**Results**

We excluded from the analyses the responses that were vague (e.g., “All that a sincere person would do can be done by a liar”). We computed a restrictiveness index based on the number of produced behaviors ($R_{b}$) for each participant and each trait. Similar to Equation (1), we calculated $R_{b}$ as the difference between the number of noncorrespondent behaviors performed by a person at the positive trait end, $N_{p}$, and the number of noncorrespondent behaviors performed by a person at the negative end, $N_{n}$:

$R_{b}= N_{p}-N_{n}$. (6)

We compared the $R_{b}$ indexes for the competence- and morality-related traits with zero, which indicates an equal number of trait-inconsistent behaviors produced for both positive and negative trait endpoints, by using one-sample *t*-tests. We used the Benjamini-Hochberg’s (1995) correction, but none of the three tests we conducted yielded a significant result. This lack of significance is probably due to the lack of power. The competence-related traits evoked an equal number of trait-inconsistent behaviors at their opposite endpoints. Indeed, the difference between the competence index (*M* = .12, *SD* = .93), 95% CI of the difference [-.32, .55], and zero was not significant, *t*(19) = .56, *p* = .580, *d* = .13. The morality-related traits tended to be perceived as asymmetric in terms of the trait-inconsistent behaviors associated with their endpoints. Participants produced a higher number of trait-inconsistent behaviors for target persons at the positive morality trait end than at the negative morality trait end (*M* = .68, *SD* = 1.4), 95% CI of the difference [.03, 1.34], but this difference was not significant when correcting for multiple comparisons using the Benjamini-Hochberg procedure, *t*(19) = 2.18, *p* = .042, *d* = .49. Finally, we compared the $R_{b}$ values of competence- and morality-related traits by means of a paired *t*-test. The difference between the three collapsed competence-related traits and the three collapsed morality-related traits, 95% CI of the difference [-1.26, .12], was not significant, *t*(19) = -1.72, *p* =.102, Cohen’s corrected *d* = .39.

**Discussion**

The results were used to select concrete trait-inconsistent behaviors to be presented to participants in Study 3. We also used these data to test a hypothesis. This study focused on the number of trait-inconsistent behaviors that participants produced for each of the two endpoints of a trait continuum. Whether or not there is a balance between the number of trait-inconsistent behaviors at the two poles of a trait might reflect the equal or different perceived behavioral ranges associated with the trait’s poles. However, the results should be interpreted with caution because the sample size was small. There was a trend in line with the hypothesized participants’ perceptions of a positive asymmetry along the morality dimension. Participants tended to produce a higher number of trait-inconsistent behaviors associated with the positive versus the negative trait pole in the case of morality-related traits. With regard to competence-related traits, we found that participants generated an equal amount of trait-inconsistent behaviors for the positive and negative poles of the traits.
