# Supplementary material for: You are fair, but I expect you to also behave unfairly: Positive asymmetry in trait-behavior relations for moderate morality information
Source: PLoS One. 2017 Jul 11;12(7):e0180686. doi: 10.1371/journal.pone.0180686 (PMC5507453; doi:10.1371/journal.pone.0180686)
Supplement: S6 Text — (DOCX) [file pone.0180686.s007.docx]

**S6 Original Version in Italian and the English Translation of the 48 Items Used in Study 3**

Quanto è frequente che una persona bugiarda dica la verità?

Quanto è frequente che una persona bugiarda riveli i propri sentimenti alla persona amata?

Quanto è frequente che una persona sincera copra qualcuno?

Quanto è frequente che una persona sincera ometta delle informazioni?

Quanto è frequente che una persona sincera dica la verità?

Quanto è frequente che una persona sincera riveli i propri sentimenti alla persona amata?

Quanto è frequente che una persona bugiarda copra qualcuno?

Quanto è frequente che una persona bugiarda ometta delle informazioni?

Quanto è frequente che una persona equa usi due pesi e due misure?

Quanto è frequente che una persona equa favorisca i propri amici a scapito di altri?

Quanto è frequente che una persona iniqua divida il conto del ristorante per il numero di persone?

Quanto è frequente che una persona iniqua tratti i clienti in egual modo?

Quanto è frequente che una persona iniqua usi due pesi e due misure?

Quanto è frequente che una persona iniqua favorisca i propri amici a scapito di altri?

Quanto è frequente che una persona equa divida il conto del ristorante per il numero di persone?

Quanto è frequente che una persona equa tratti i clienti in egual modo?

Quanto è frequente che una persona retta violi il codice della strada?

Quanto è frequente che una persona retta sparli di una persona?

Quanto è frequente che una persona scorretta paghi il biglietto sui mezzi pubblici?

Quanto è frequente che una persona scorretta paghi le tasse?

Quanto è frequente che una persona scorretta violi il codice della strada?

Quanto è frequente che una persona scorretta sparli di una persona?

Quanto è frequente che una persona retta paghi il biglietto sui mezzi pubblici?

Quanto è frequente che una persona retta paghi le tasse?

Quanto è frequente che una persona competente vada in confusione?

Quanto è frequente che una persona competente si dimentichi qualcosa?

Quanto è frequente che una persona incompetente usi parole tecniche o parli forbito?

Quanto è frequente che una persona incompetente mostri sicurezza?

Quanto è frequente che una persona incompetente vada in confusione?

Quanto è frequente che una persona incompetente si dimentichi qualcosa?

Quanto è frequente che una persona competente usi parole tecniche o parli forbito?

Quanto è frequente che una persona competente mostri sicurezza?

Quanto è frequente che una persona efficiente impieghi troppo tempo per portare a termine un lavoro?

Quanto è frequente che una persona efficiente si perda nei dettagli?

Quanto è frequente che una persona inefficiente rispetti il proprio orario di lavoro e le consegne?

Quanto è frequente che una persona inefficiente si impegni per portare a termine un lavoro?

Quanto è frequente che una persona inefficiente impieghi troppo tempo per portare a termine un lavoro?

Quanto è frequente che una persona inefficiente si perda nei dettagli?

Quanto è frequente che una persona efficiente rispetti il proprio orario di lavoro e le consegne?

Quanto è frequente che una persona efficiente si impegni per portare a termine un lavoro?

Quanto è frequente che una persona intelligente metta in atto azioni imprudenti?

Quanto è frequente che una persona intelligente faccia scherzi esagerati a colleghi e amici?

Quanto è frequente che una persona stupida faccia scelte di vita adeguate?

Quanto è frequente che una persona stupida impari dai propri errori?

Quanto è frequente che una persona stupida metta in atto azioni imprudenti?

Quanto è frequente che una persona stupida faccia scherzi esagerati a colleghi e amici?

Quanto è frequente che una persona intelligente faccia scelte di vita adeguate?

Quanto è frequente che una persona intelligente impari dai propri errori?

How often does a liar tell the truth?

How often does a liar disclose their feelings to the one they love?

How often does a sincere person cover for somebody?

How often does a sincere person omit some information?

How often does a sincere person tell the truth?

How often does a sincere person disclose their feelings to the one they love?

How often does a liar cover for somebody?

How often does a liar omit some information?

How often does a fair person have double standards?

How often does a fair person favor their friends at the expense of others?

How often does an unfair person divide the restaurant’s bill by the number of people?

How often does an unfair person treat the customers equally?

How often does an unfair person have double standards?

How often does an unfair person favor their friends at the expense of others?

How often does a fair person divide the restaurant’s bill by the number of people?

How often does a fair person treat the customers equally?

How often does a righteous person breach the traffic laws?

How often does a righteous person speak ill of somebody?

How often does an unrighteous person pay the ticket of public transport?

How often does an unrighteous person pay taxes?

How often does an unrighteous person breach the traffic laws?

How often does an unrighteous person speak ill of somebody?

How often does a righteous person pay the ticket of public transport?

How often does a righteous person pay taxes?

How often does a competent person get confused?

How often does a competent person forget something?

How often does an incompetent person use technical jargon or speak in a polished way?

How often does an incompetent person exhibit self-confidence?

How often does an incompetent person get confused?

How often does an incompetent person forget something?

How often does a competent person use technical jargon or speak in a polished way?

How often does a competent person exhibit self-confidence?

How often does an efficient person take too much time to complete work?

How often does an efficient person lose themselves in details?

How often does an inefficient person respect their working hours and instructions?

How often does an inefficient person commit to completing work?

How often does an inefficient person take too much time to complete work?

How often does an inefficient person lose themselves in details?

How often does an efficient person respect their working hours and instructions?

How often does an efficient person commit to completing work?

How often does an intelligent person take ill-advised actions?

How often does an intelligent person make exaggerated jokes to colleagues and friends?

How often does a stupid person make adequate life choices?

How often does a stupid person learn from their mistakes?

How often does a stupid person take ill-advised actions?

How often does a stupid person make exaggerated jokes to colleagues and friends?

How often does an intelligent person make adequate life choices?

How often does an intelligent person learn from their mistakes?
