# Supplementary material for: You are fair, but I expect you to also behave unfairly: Positive asymmetry in trait-behavior relations for moderate morality information
Source: PLoS One. 2017 Jul 11;12(7):e0180686. doi: 10.1371/journal.pone.0180686 (PMC5507453; doi:10.1371/journal.pone.0180686)
Supplement: S7 Text — (DOCX) [file pone.0180686.s008.docx]

**S7 Analyses of Trait-Inconsistent Behavior Frequency in Study 3 with the Cue-Validity Index**

Although the *R* index expressed in Equation (1) seems a reasonable and adequate dependent variable, we tested whether our results held also with a different dependent variable that has already been used in the literature. We thus divided by 10 the 0-10 participants’ estimates so that we obtained probability values and then we computed the normalized proportion used by Skowronski and Carlston (i.e., the “cue-validity index”, Skowronski & Carlston, 1987, p. 692), which is expressed by the following equation:

$B=\frac{p\left( D/H \right)}{\left( p\left( D/H \right)+p\left( D/{\neg H} \right) \right)}$. (7)

Where, *D* stands for the behavior (e.g., telling the truth), *H* represents the target person with the trait (e.g., sincere) or its opposite (e.g., insincere). This index varies between 0 and 1. A value of .5 indicates that the behavior under consideration is equally likely under the two poles of the trait. A value > .5 indicates that the behavior is more linked to *H* than to $\neg H$, whereas a value < .5 means that the behavior is more linked to $\neg H$ than to *H*. Furthermore, with this index, the value relative to one disposition (e.g., sincere) would be the complement of the value obtained for its opposite (e.g., sincere). For example, suppose that *H* stands for a sincere person (and thus $\neg H$ for an insincere person), and *D* is “telling the truth”. If a participant estimates that the frequency of a sincere person telling the truth is 9 (on a 0-10 scale), and that an insincere person telling the truth is 2, then the index $B_{c}$ indicating to what extent “telling the truth” is related to being sincere would be:

$B_{c}=\frac{.9}{\left( .9+.2 \right)}=.82$ (8) Conversely, the index $B_{n}$ indicating the relationship between “telling the truth” and being insincere would be:

$B_{n}=\frac{.2}{\left( .2+.9 \right)}=.18$ (9)

Accordingly, “telling the truth” would be perceived as more related to sincere persons than to insincere persons.

We computed $B_{n}$, that is, the index relative to trait-inconsistent behaviors, for positive and negative competence- and morality-related traits. The reliabilities were overall satisfactory, with Cronbach’s alphas ranging from .62 to .73 for moral, immoral, competent and incompetent trait-inconsistent behaviors. Therefore, we computed average scores for each pole of each dimension. We then subtracted the average scores for the negative trait poles (i.e., incompetence and immorality) from the average scores for the positive trait poles (i.e., competence and morality). We then conducted three *t*-tests and, for each of them, we used adjusted significance levels of .0167 following Benjamini and Hochberg’s (1995) correction. We compared these differences with zero by means of a one-sample *t*-test. We found that the difference between the positive and negative poles was significantly higher than zero for the morality dimension (*M* = .03, *SD* = .07), 95% CI of the difference [.02, .05], *t*(87) = 4.06, *p* < .001, *d* = .43, whereas it was not significant for the competence dimension (*M* = .02, *SD* = .08), 95% CI of the difference [-.00, .03], *t*(87) = 1.73, *p* = .087, *d* = .18. The difference between the morality and competence indexes (95% CI of the difference [-.01, .04]) was not statistically significant, *t*(87) = 1.45, *p* = .150, Cohen’s corrected *d* = .10.
